# Squamation and ecology of thelodonts

**DOI:** 10.1371/journal.pone.0172781

**Published:** 2017-02-27

**Authors:** Humberto G. Ferrón, Héctor Botella

**Affiliations:** Institut Cavanilles de Biodiversitat i Biologia Evolutiva, Paterna, Valencia, Spain; University of Michigan, UNITED STATES

## Abstract

Thelodonts are an enigmatic group of Paleozoic jawless vertebrates that have been well studied from taxonomical, biostratigraphic and paleogeographic points of view, although our knowledge of their ecology and mode of life is still scant. Their bodies were covered by micrometric scales whose morphology, histology and the developmental process are extremely similar to those of extant sharks. Based on these similarities and on the well-recognized relationship between squamation and ecology in sharks, here we explore the ecological diversity and lifestyles of thelodonts. For this we use classic morphometrics and discriminant analysis to characterize the squamation patterns of a significant number of extant shark species whose ecology is well known. Multivariate analyses have defined a characteristic squamation pattern for each ecological group, thus establishing a comparative framework for inferring lifestyles in thelodonts. We then use this information to study the squamation of the currently described 147 species of thelodonts, known from both articulated and disarticulated remains. Discriminant analysis has allowed recognizing squamation patterns comparable to those of sharks and links them to specific ecological groups. Our results suggest a remarkable ecological diversity in thelodonts. A large number of them were probably demersal species inhabiting hard substrates, within caves and crevices in rocky environments or reefs, taking advantage of the flexibility provided by their micromeric squamations. Contrary to classical interpretations, only few thelodonts were placed among demersal species inhabiting sandy and muddy substrates. Schooling species with defensive scales against ectoparasites could be also abundant suggesting that social interactions and pressure of ectoparasites were present in vertebrates as early the Silurian. The presence of species showing scales suggestive of low to moderate speed and a lifestyle presumably associated with open water environments indicates adaptation of thelodonts to deep water habitats. Scale morphology suggests that some other thelodonts were strong-swimming pelagic species, most of them radiating during the Early Devonian in association with the Nekton Revolution.

## Introduction

Thelodonts are an extinct group of agnathan fishes that ranged from the Upper Ordovician [1, 2, 3] to the Upper Devonian [4, 5] being one of the oldest vertebrate clades in the fossil record. Thelodonts differ from all other extinct Paleozoic jawless fishes in the structure of their exoskeleton. Instead of compact dermal armor plates and/or large flat scales present in pteraspidomorphs, galeaspids, pituriaspids, osteostracans and anaspids, the body of thelodonts was covered by characteristic micromeric dentine placoid-like scales. Due to the abundance and ubiquity of these remains, thelodonts have been extensively studied from biostratigraphical, paleogeographical and taxonomic points of view (e.g. [3, 6, 7, 8, 9, 10] and references therein). Nevertheless, the knowledge of their ecology and mode of life is still scarce due to a rarity of articulated remains, as well as an apparent lack of close extant relatives that might be investigated as modern analogues.

In fact, previous interpretations on the habitats of thelodonts are funded basically on sedimentological data (See [3] and references therein). Most species of this group have been found in sediments representing a wide range of marine environments, including shallow-water lagoons (e.g. *Phlebolepis elegans* from the Paadla Stage on western Saaremaa, Estonia), near shore habitats (e.g. thelodonts from the Canadian Artic) and open shelf or even deep-water basins (e.g. thelodonts from some sections of the East Baltic region). In addition, a few species have been found in sediments interpreted by some authors as brackish or freshwater (e.g. *Turinia pagei* from the Lower Devonian Old Red Sandstone facies sediments of Scotland and the Anglo-Welsh cuvette). Turner [11] inferred the lifestyle of some species of thelodonts on the basis of morphological and anatomical evidence suggesting that thelodonts were adapted both to pelagic and benthic habitats. Later, Turner [12] made a compilation of the thelodont localities known up to date and described the Silurian and Devonian thelodont communities, noting their taxonomic composition, age and the environment they came from, providing thus an overview about the plausible diversity of habitats that thelodonts occupied.

Here we present a novel approach for investigating the ecological diversity of thelodonts based on the relationships between scale morphology and ecology observed in extant fishes. In this sense the close correspondence between squamation pattern and lifestyle of extant sharks is well known and has been extensively studied [13, 14, 15, 16, 17, 18, 19, 20, 21, 22]. Interestingly, the body surface of sharks is covered almost entirely with micromeric dentinous placoid scales as in thelodonts [23, 24, 25]. Several authors have noted the similarities in the morphology and the histology as well as in the developmental process in thelodont scales and those found in neoselachians and some pre-Carboniferous chondrichthyans [23, 24, 26, 27]; being the only two obvious differences the growing base with complex roots and the absence of neck vascular canals [28, 29]. Similarities are so apparent that thelodont scales were classified for some time as shark placoid scales and thelodonts included within selachians (e.g. [30, 31, 32, 33, 34, 35, 36, 37]). Based on the high similarity between scales of both groups several authors have noted that they can be understood as functional analogs and the functions of thelodont scales should have been essentially the same that those present in modern sharks [3, 25, 27, 28, 38]. Therefore we propose that a detailed analysis of the relationships between squamation patterns and ecology of extant sharks can provide a comparative framework for the study of paleoecology and lifestyles of thelodonts.

### Diversity and squamation of thelodonts

Thelodonts are a rather morphologically diverse group, comprising species with dorso-ventrally flattened body, broad head and hypocercal large asymmetrical tails; species with fusiform body and similar tails; and species with laterally flattened body and strong multilobed fork-shaped tails. The first two morphologies are found in species that have been referred to “conventional” (= non-furcacaudiform) thelodonts whereas the latter is typical of furcacaudiform thelodonts. Paired pectoral flaps (pectoral fins?) are generally present in thelodonts, although they might have been absent in some (or all) furcacaudiforms. Dorsal and anal fins have been recognized in some non-furcacaudiform thelodonts, but all furcacaudiforms appear to lack the latter. Moreover, a pair of ventral flaps (pelvic fins?) is known in some species of fork-tailed thelodonts and in one shieliform. The last extended overview of general morphology and diversity of the groups was given by Märss et al. [3]. After that only seven new species have been described [5, 39, 40, 41].

Currently, there are 147 described thelodont species, belonging to 54 different genera and grouped in six orders (Sandiviiformes, Loganelliiformes, Shieliiformes, Phlebolepidiformes, Thelodontiformes and Furcacaudiformes). Only 29 of these species are known from articulated specimens, which provide the information about the general aspect and some anatomical features of thelodonts. The remaining 118 species are described only on the basis of associations of (or a few) disarticulated scales. Nevertheless, the well-accepted criteria for defining thelodont species based on associations of isolated scales (see [7, 28, 38, 42, 43, 44]) allows paleoichthyologists to differentiate not only morphospecies but also purported biological species [45]. These criteria include the necessity of recognizing morphological variability present in scales of different body areas of the same individual. Such variability has been extensively analyzed from articulated specimens by several authors and summarized in series or “topological” types of scales. Gross [42], identified three series of scales for non-furcacaudiform thelodonts (head, transitional and trunk scales). Subsequently Märss [46] distinguished up to five distinct series (rostral, cephalo-pectoral, postpectoral, precaudal and pinnal scales). These classifications are comparable as follows: head scales are equivalent to oral/rostral scales, transitional scales are equivalent to cephalo-pectoral scales and trunk scales are equivalent to postpectoral, pinnal (fins) and precaudal scales [38]. In addition, some other scale morphological variations have been described in different body areas, such as the orbital scales and orbital platelets [47, 48, 49], specialized scales from the pharyngeal, branchial and extrabranchial regions [3, 47, 50, 51, 52, 53, 54] and the scales of the fin leading and training edges [25, 38, 47, 55, 56, 57]. In furcacaudiform thelodonts Wilson and Caldwell [54, 58] and Caldwell and Wilson [59] described four series of scales (preorbital, head/branchial, flank/tail and dorsal/ventral median ridge scales).

Thus, the squamation of thelodonts is a highly complex structure composed of micromeric scales the morphology of which varies not only interspecifically but also within the same species depending on the body area. In addition, some studies evidenced the presence of ontogenetic variation in the squamation of several thelodont species [11, 28, 47, 60] and possible sexual dimorphism [11]. Therefore, it is obvious that the squamation had to play an essential role in the ecological diversity of the group.

### Squamation and ecology of sharks

The presence of similar scale morphologies and squamation patterns in phylogenetically distant shark species but with similar lifestyles evidences that placoid scales display great plasticity and potential for adaptation to different ecological conditions [13, 14, 15]. Scales of the selachians are involved in at least four different functions: protection against abrasion, defense against ectoparasites and the settlement of epibionts, reduction of the skin friction drag and accommodation of photophores in bioluminescent sharks [14, 15]. Placoid scales can be specialized in at least one of these four functions or can be a trade-off between more than one of them (i.e., scales with generalized functions). Reif [15] summarized the morphological variability present in the placoid scales into at least ten characteristic morphologies assigning each of them to one functional type. Furthermore, he analyzed the relationship between the squamation and ecology in living sharks. For this he performed an ecological classification of current sharks based mainly on their habitat preferences and distinguished the predominant functional types of scales of each ecological group.

Reif´s [15] classification consists of the following seven ecological groups: (1) large near-shore hunters, (2) fast pelagic hunting species, (3) schooling species of low to moderate speed, (4) demersal species on rocky substrates and in caves, (5) demersal species on sandy and muddy substrates, (6) mesopelagic luminescent species and (7) slow species of the open water. According with Reif [15], abrasion resistant scales are found in species living close to the bottom (i.e., demersal sharks on hard substrates and demersal sharks on sandy and muddy substrates), although they may be present also in other ecological groups in small areas subjected to an abrasive stress (e.g. mouth, snout or leading edge of the fins). These scales have knob-shaped crowns with either smooth or strongly ornamented surface and frequently show scratch marks. Scales with defensive functions against ectoparasites and the settlement of epibionts are common in demersal sharks inhabiting muddy or sandy substrates, in schooling sharks of low to moderate speed, and in slow sharks of the open water. These scales are spine-like with the main cusp pointing in an upward-posterior direction usually surrounded by mucus produced by well-developed glands in the skin. Drag reduction scales are predominant among fast pelagic hunting species and large near-shore hunters. Scales of this functional type have ridges or riblets aligned in the direction of fluid flow, narrowly spaced (35–80 μm) in fast pelagic hunting sharks and wider spaced (more than 80 μm) in large near-shore hunters. Scales related to bioluminescence have evolved in some mesopelagic sharks, enabling the skin to carry photophores and permitting light to pass between them [61]. These scales can show different morphologies including large crowns with concave facets, bristle-like crowns or thorn-like or hook- like crowns varying in density depending on the species. Scales of generalized functions are present in several of the ecological groups. These scales have a number of long ridges, in some cases converging at the top of the crown, and usually well-developed lateral wings. This information is summarized in Table 1.

**Table 1:** Description of the scale characteristic morphologies and functional types distinguished by Reif [15, 61], and their equivalence with the scale morphotypes considered in the present study.

Reif's functional type	Reif's characteristic morphology	Morphotype in this work	Examples of taxa showing each scale morphotype
Accommodation of photophores	Scales with large crowns and concave facets	M. 1	*Dalatias licha*, *Isistius brasiliensis*, *Etmopterus schultzi* and *Chlamydoselachus anguineus*
Accommodation of photophores	Scales with long bristle-like crowns	M. 2	*Etmopterus spinax*
Accommodation of photophores	Scales with hook-like crowns	M. 3	*Etmopterus bullisi*, *E*. *hillianus*, *E*. *unicolor* and *E*. *lucifer*
Accommodation of photophores	Scales with thorn-like crowns widely spaced	M. 3	*Centroscyllium fabricii*, *C*. *ritteri* and *Etmopterus virens*
Defense against ectoparasites and epibionts	Scales with spine-like crown with the main cusp pointing in an upward-posterior direction	M. 4	*Squalus* and *Deania*
Drag reduction	Scales with parallel long ridges with an average distance of 35–80 μm. This distance is constant in all scales.	M. 5	*Isurus*, *Lamna*, *Carcharodon*, *Sphyrna*, some *Carcharinus* spp., among others
Drag reduction	Scales with parallel long ridges with an average greater than 80 μm. This distance varies between scales.	M. 5	*Galeocerdo*, some *Carcharhinus spp*., *Negraprion*, *Triaenodon*, *Odontaspis*, among other genera
Protection against abrasion	Scales with large and thickened smooth crown	M. 6	Most demersal sharks. Also on the snout, surronding area of the mouth and leading edges of fins of all sharks
Protection against abrasion	Scales with large and thickened ornamented crown	M. 7	*Heterodontus* and *Centrophorus*
Generalized functions	Scales with long ridges and usually well developed lateral wings	M. 8	*Mustelus*, Family Scyliorhinidae, Family Hexanchidae

Other atypical functions has also been documented, including scales specialized in hatching in newborns of *Cephaloscyllium ventriosum* [65] or the use of placoid scales in prey processing in *Scyliorhinus canicula* [66]. However these are considered as specific adaptations and the general classification into the functional types proposed by Reif [14, 15] is well established and has been followed in subsequent studies (e.g. [13, 16, 17, 18, 19, 20, 67, 68, 69, 70]).

Here we characterize scale characteristic morphologies proposed by Reif [14, 15] using morphometrics and discriminant analysis and studied the squamation patterns of a significant number of extant shark species. Subsequently, we have further analyzed the relationship between the squamation patterns and ecology in extant sharks establishing a comparative framework for inferring lifestyles in fossil groups with similar squamations whose ecology is poorly understood. Finally, with the aim of providing an overview of the lifestyles and ecology of thelodonts, we have studied their squamation patterns from both articulated and disarticulated remains and compared them quantitatively and qualitatively with those described in each ecological group of sharks.

## Material

The squamation patterns of 56 specimens belonging to 53 different species of sharks were analyzed. Taxa were selected trying to span the phylogeny of the group, being represented seven of the nine extant orders, and covering a wide range of the lifestyles of sharks. The studied specimens come from the collections of (1) Museu Cau del Tauró de l’Arboç (MCTA, Spain), (2) Museu de Zoologia de Barcelona (MZB, Spain), (3) Museu Oceanográfico do Vale do Itajaí (MOVI, Brasil) and (4) Museum für Naturkunde (ZMB, Germany) and (5) commercial fisheries from the western Mediterranean coast. Additionally, skin fragments of two more species from the MCTA collection (*Centrophorus granulosus* and *Oxynotus centrina*) were studied. Permission to work with specimens was granted by the MCTA (including specimens of the MOVI), MZB and ZMB. The registration numbers, sex and length of all studied specimens are available in S1 Table.

The squamation patterns of thelodonts were analysed in all the currently described species of the group. Data were obtained essentially from literature, where scales of all thelodont species are well described and figured. In the case of species erected on the basis of disarticulated elements, accurate descriptions of scales are given as a result of the lack of other more complete remains; whereas in the case of species known by articulated squamations detailed descriptions are given in order to determine the topological scale variability that can occur in one specimen for accurate identification of species from disarticulated assemblages. Complementarily, 24 articulated specimens belonging to six different species have been studied first hand in the collections of the National Museum of Scotland (NMS.G., Edinburgh, Scotland) and the Museum für Naturkunde (MB.f., Berlin, Germany) (S2 Table).

The preservation style of the 29 species known from articulated remains is diverse. Accordingly, 14 species are known from complete or nearly complete squamations, seven from partial squamations and three from patches of scales. Additionally, five complete reconstructions generated from several incomplete articulated remains were also studied. The exposed area depends largely on the body shape. In dorso-ventral flattened species the specimens usually expose the dorsal or ventral part of the body with the tail in lateral view; however the identification of which side of the animal is visible is sometimes difficult because of poor preservation. Complete squamations therefore, can only be studied in species with specimens preserved in dorsal and ventral views. In furcacaudiform thelodonts the preservation is usually in lateral view, due to their apparent lateral flattening. In consequence, the possible dorso-ventral variation of squamation is represented, which allows the study of the complete squamation. References, registration numbers and details of preservation of all studied articulated specimens are given in S3 Table.

Institutional abbreviations: AM.F., Australian Museum (Australia); BMNH, British Museum of Natural History (United Kingdom); GSC, Geological Survey of Canada (Canada); GSE, British Geological Survey Museum (deposited at NMS.G.) (United Kingdom); MB.f., Museum für Naturkunde (Germany); MCTA, Museu Cau del Tauró de l’Arboç (Spain); MCZ, Museum of Comparative Zoology (United States); MOVI, Museu Oceanográfico do Vale do Itajaí (Brasil); MZB, Museu de Zoologia de Barcelona (Spain); NMC, Canadian Museum of Nature (Canada); NMS.G., National Museum of Scotland (United Kingdom); Pi, Institute of Geology (Estonia); TUG, Museum of Geology (Estonia); UALVP, University of Alberta Laboratory for Vertebrate Paleontology (Canada); ZMB, Museum für Naturkunde (Germany).

## Methodology

### Analysis of the squamation patterns in extant sharks

#### Morphometric characterization of placoid scales

Scale characteristic morphologies proposed by Reif [14, 15] were characterized using classical morphometrics and discriminant analysis (see by example [71]). As most of the described species of thelodonts are known only from isolated scales, we selected only variables measurable in single scales whereas other characters concerning measurements on a set of scales (i.e., density, coverage or scale morphological variation within a single specimen) were discarded from this study. Hence, two characteristic morphologies of scales related to bioluminescence, which were determined by Reif [15] essentially based on the coverage density, were unified into a single morphotype here. In addition, as discriminant analysis does not tolerate missing data, variables not measurable in all scales were also discarded. Hence, the two characteristic morphologies of scales with drag reduction functions, which were differentiated by Reif [15] based on the distance between ridges and the variability of this character within the same species, were also combined into one morphotype. However, an adittional morphometric analysis on the thelodonts scales classified into this morphotype has been implemented a posteriori in order to determine which of the Reif´s original drag reduction morphologies they belong to (see below). In sum, the ten characteristic morphologies differentiated by Reif [14, 15] were here synthesized into eight morphotypes (See Table 1).

For the morphometric characterization, a number of scales of each morphotype were chosen among the studied individuals and photographed in dorsal view. The selection was based on the work of Reif [15] and realized in such a way as to obtain broad taxonomic representation. A total of ten variables were measured on the crown of each scale, some of which were subsequently combined to generate new adimentional variables (Table 2). A discriminant analysis (Canonical Variate Analysis, CVA-1) was performed from the obtained morphometric data. We took as defined groups the eight morphotypes and included as discriminating variables circularity, roundness, solidity and those obtained by combining the original variables (Table 2). The measurements were taken with ImageJ software 1.46r and CVA was carried out using PASW Statistics 18 software.

**Table 2:** Code designations and description of the variables measured on the crown surface of shark scales and the size-free variables included in the Canonical Variate Analysis-1 (CVA-1).

Measured variables	Variables in the analysis
MLC (Maximum Length of Crown)	MLC/MWC
MWC (Maximum Width of Crown)	RLA/MLC
RLA (Ridge Lenght Average)	RLA^2^/ACS
LLR (Length of the Longest Ridge)	(MLC*MWC)/ACS
LSR (Length of the Shortest Ridge)	LLR/MLC
ACS (Area of the Crown Surface)	(LLR-LSR)/MLC
CP (Crown Perimeter)	CP/MLC
Circularity [4*π*(ACS/CP^2^)]	Circularity
Roundness [4*ACS/(π*Major axis^2^)]	Roundness
Solidity (ACS/Convex area)	Solidity

#### Quantification of the body surface area covered by each scale morphotype in all species

**Fig 1:**
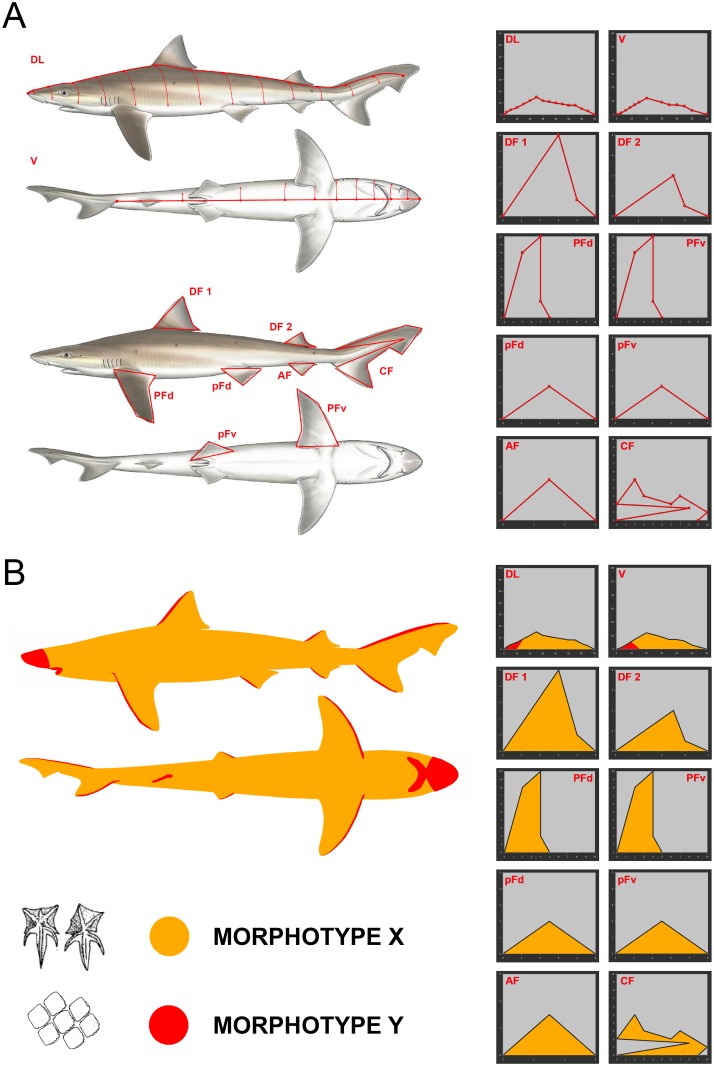
Diagram illustrating the methodology used to quantify the body surface area covered by each scale morphotype in a model shark (*Galeorhinus galeus*). (A) Representation of the different body areas on Cartesian coordinate systems. (B) Hypothetical body distribution of the scale morphotypes and their representation on the Cartesian coordinate systems (scale morphotypes covering negligible surfaces, such as the circum-oral region or the fin leading edges, have not been considered for the quantitative approach and are not shown on the graphs). *Galeorhinus galeus* drawing taken from Shark Trust [72], courtesy of Marc Dando. Scale drawings modified from Compagno [62]. Abbreviations: AF, anal fin; CF, caudal fin; DF 1, first dorsal fin; DF 2, second dorsal fin; DL, dorso-lateral region; PFd, dorsal surface of the pectoral fin; pFd, dorsal surface of the pelvic fin; PFv, ventral surface of the pectoral fin; pFv, ventral surface of the pelvic fin; V, ventral region.

The body of sharks was virtually divided into several regions (dorso-lateral, ventral, and fins) whose areas were represented separately on Cartesian coordinate systems (Fig 1A). Once the body surface of each specimen was modeled, a visual inspection under binocular microscope was performed. All morphological variants present in each specimen were noticed (establishing the boundaries between them), photographed and morphometrically characterized. Scales were then introduced into the CVA-1, being assigned to one of the eight morphotypes. This allowed us to represent the boundaries between morphotypes in the Cartesian graphs and calculate the area covered by each morphotype for all studied specimens (Fig 1B). Measurements were performed with ImageJ 1.46r software. Scales of some body regions of particular interest but covering negligible area, e.g. circum-oral region, nictitating membranes, claspers or fin leading edges, were also photographed and considered qualitatively but not quantitatively.

A binocular microscope LEICA MS5, cold light source with optical fiber LEICA CLS 150X, Leica DFC 420 digital camera and image capture software LEICA APPLICATION SUITE 4.0.0 were used for the study of specimens deposited at the Museu Cau Arboç Tauró and the Museum für Naturkunde of Berlin and those obtained from the Fishermen's Association of Burriana (Spain), which were later discarded. Binocular microscope Motic—SMZ 168, light source LED-60T, 2500 Moticam digital camera and image capture software Motic images Plus 2.0 were used for specimens deposited at the Museum of Zoology of Barcelona.

Relationship Between Squamation Pattern and Lifestyle In Sharks.

#### Establishment of ecological groups and placement of the studied species within them

**Fig 2:**
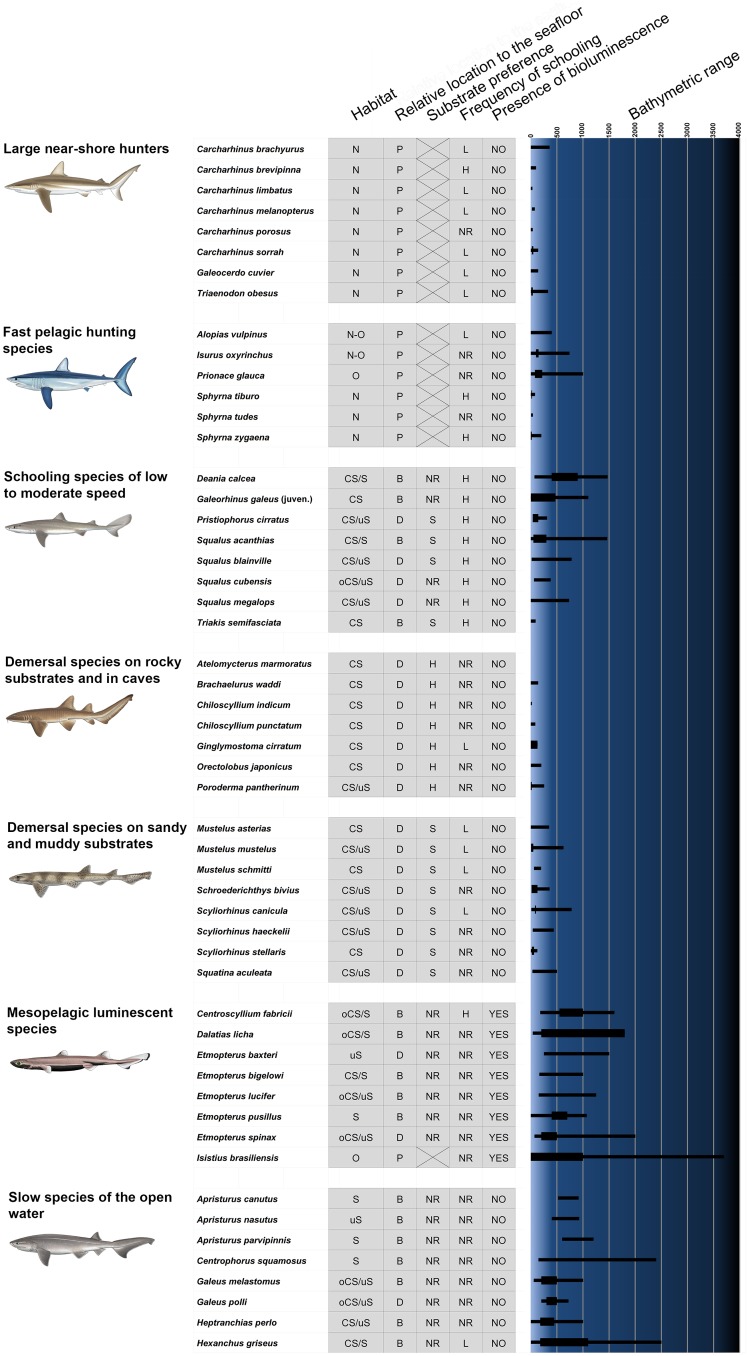
Ecological parameters considered for all studied species of sharks arranged by the ecological groups defined by Reif [15]. Data were mainly taken from Reif [15], Compagno [62, 63, 64], Shark Trust [72], Compagno et al. [73], Froese and Pauly [74], Ebert et al. [75] and IUCN [76]. Reported bathymetric ranges are represented by black bars where usual bathymetric ranges are indicated with thickened portions on the right side of the diagram. All shark drawings taken from Shark Trust [72], courtesy of Marc Dando. Abbreviations: Habitat (CS, continental shelf; N, neritic zone; O, oceanic zone; oCS, outer continental shelf; S, slope; uS, upper slope), relative location to the seafloor (B, benthopelagic; D, demersal; P, pelagic), substrate preference (H, hard substrate; NR, not reported; S, soft substrate), frequency of schooling (H, high; L, low; NR, not reported).

The habitat, reported bathymetric range, usual bathymetric range, relative location to the seafloor (demersal, benthopelagic or pelagic), substrate preference (soft or hard), presence of bioluminescence and frequency of schooling (low or high) were determined for all the 53 species of sharks analyzed in this study. Data were mainly taken from Reif [15], Compagno [62, 63, 64], Shark Trust [72], Compagno et al. [73], Froese and Pauly [74], Ebert et al. [75] and IUCN [76]. Based on these parameters the studied species were included in one of the seven ecological groups proposed by Reif [15] (Fig 2). The update of ecological parameters required the relocation of some species into a different ecological group with respect to the original work of Reif [15]. *Ginglymostoma cirratum* was transferred from demersal species on sandy and muddy substrates to demersal species on rocky substrates and in caves given its preference for hard substrates [74, 75, 76]; *Centrophorus squamosus* and *Galeus melastomus* were relocated from demersal species on sandy and muddy substrates to slow species of the open water given that their bathymetrical ranges and benthopelagic habits are more in agreement with those of the second ecological group [75, 77]; and finally, the *Galeorhinus galeus* juvenile was included in schooling species of low to moderate speed because of differences from the adult lifestyle [74, 75, 76].

#### Relationship between squamation pattern and lifestyle

The relationship between the squamation pattern and the lifestyle in extant sharks was analyzed in two ways. First, we performed a quantitative treatment of body coverage data of scale morphotypes by a new Canonical Variate Analysis (CVA-2) taking as defined groups six ecological groups (modified from Reif [15]) and including as discriminant variables the percentages of coverage of each scale morphotype. Ecological groups of fast pelagic hunting species and large near-shore hunters were combined into a single one (strong-swimming pelagic species) since Reif [15] differentiated them on the basis of two distinct drag reduction scale morphologies that have been unified here into a unique morphotype (see above). Secondly, we described and compared qualitatively the typical squamation of each ecological group, taking into account relevant aspects such as the distribution of each scale morphotype on the body or the presence of certain scales occupying negligible body areas. CVA was carried out using PASW Statistics 18 software.

### Analysis of squamation patterns in thelodonts

#### Thelodont species known from articulated squamations

Once the comparative framework was established with extant sharks, the squamation patterns of the 29 thelodonts species known from articulated squamations were analyzed both quantitatively and qualitatively. Scale morphological variants were identified in each specimen and their body coverage areas were delimited and quantified using Adobe Photoshop CS3 Extended and ImageJ 1.46r. Next, each morphological variant was assigned to one morphotype (and one functional type) of those defined for placoid scales including them in the CVA-1. This made it possible to calculate the percentage of body area cover of each morphotype and functional type in all species. Subsequently all specimens were included in the discriminant analysis CVA-2 performed on sharks, allowing their assignments to one ecological group. However, as it has been noted above, the nature of specimens preserved in different species is diverse, including species known from specimens in ventral view, others from specimens in dorsal view and others from specimens in lateral view. Thus, in order to maximize the number of articulated thelodont species included in the multivariate analysis, some variants of the CVA-2 were implemented. We performed three different CVAs with data from extant sharks taking as defined groups the six ecological groups and taking as discriminant variables the coverage percentages of scale morphotypes on the whole body (CVA-2.1, equivalent to the original analysis), only on the dorsal side of the body (CVA-2.2), or only on the ventral side of the body (CVA-2.3). In this manner, not only thelodont species known from complete squamations but also those known only from squamations in dorsal or ventral views could be included in the analysis. In those species where both dorsal and ventral squamation are known from the same individual (i.e., *Loganellia scotica*, *Shielia taiti* and *Lanarkia horrida*) the coverage areas of each scale morphotype were also calculated for the whole body and included in CVA-2.1 as a single case, although dorsal and ventral squamation patterns were also analyzed independently in CVA-2.2 and CVA-2.3. Species known only from partial squamations or patches of scales were interpreted only qualitatively.

#### Thelodont species known from disarticulated remains

**Fig 3:**
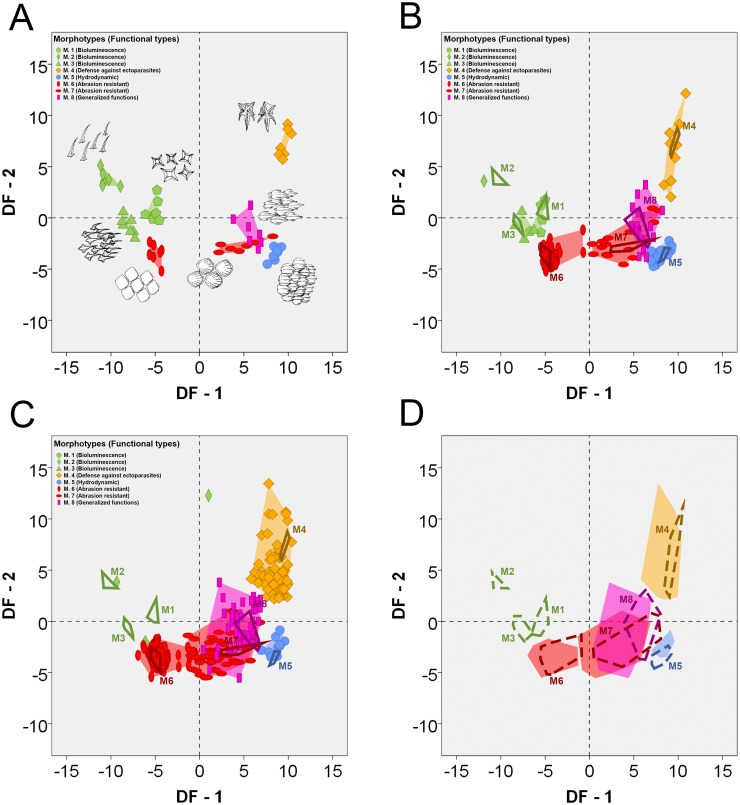
Canonical Variate Analysis-1 (CVA-1) taking eight scale morphotypes (M1-M8, corresponding to five functional types) as defined groups (modified from Reif [15]) and ten size-free variables of the crown surface as discriminant variables. Results are plotted based on the first two discriminant functions. (A) CVA-1 results of shark scales of known morphotype (original cases). (B) Classification results and discriminant punctuations of shark scales of unknown morphotype after their inclusion in CVA-1. (C) Classification results and discriminant punctuations of all studied thelodont scales after their inclusion in CVA-1 (note that all cases assigned to M.2, M.3 and M.5 come from disarticulated remains). Polygon outlines in B and C represent discriminant punctuations of original shark scales. (D) Comparison between discriminant punctuations of all shark scales (dashed polygon outlines) and all thelodont scales (filled polygons) arranged by morphotypes. Scale drawings modified from Compagno [62, 63, 64].

In the 124 species known from isolated scales (six of them being also known from articulated remains), scales of each topological series (following the original descriptions and assignments, see S4 Table) were measured and assigned to one of the morphotypes and functional types defined for placoid scales of sharks after their inclusion in CVA-1. Subsequently, with the purpose of obtaining statistically tractable data, percentages of surfaces occupied by each scale series in these species were extrapolated based on the reconstructions of Märss and Ritchie ([47]: Fig 11) and Wilson and Caldwell ([54]: Fig 3), taking *Loganellia scotica* and *Furcacauda fredholmae* as models for non-furcacaudiform and furcacaudiform thelodonts respectively. Therefore, for non-furcacaudiform thelodonts it was estimated that head, transitional and trunk scales cover the 4.6%, 22.7% and 72.7% of the body surface respectively; and rostral, cephalo-pectoral, postpectoral, precaudal and pinnal scales cover the 4.6%, 22.7%, 33.8%, 31.6% and 7.3% of the body surface respectively. For furcacaudiform thelodonts it was estimated that preorbital, head/branchial, and flank/tail scales cover the 2.6%, 12.6% and 84.8% of the body surface respectively.

In this way, percentages of the body surface covered by each scale morphotype and functional type were obtained for thelodont species known from disarticulated squamations. Data were included in the second CVA performed on extant sharks (CVA-2) assigning each thelodont species to one ecological group. However, not all the 124 species could be included in the analysis because some of them are described only from scales of unknown body position, others lack some topological series and others have more than one functional type in some topological series. Notwithstanding this, a qualitative interpretation of the morphotypes and functional types they present made possible their assignments to one ecological group in some cases (see Discussion).

Additionally, in those species of thelodonts known from both associations of isolated scales and articulated specimens, disarticulated remains were analyzed independently to provide additional support for assignments based on articulated specimens and/or additional evidences to solve possible uncertain assignments.

Finally, in order to obtain further ecological information about the species with scales that were classified into the drag reduction functional type, an additional morphometric analysis was performed. For this, we have applied the same methodology established by Reif [15] for distinguishing between scales of large near-shore hunters and fast pelagic hunting species. Accordingly, (1) we have measured the average ridge distance (ARD) and crown width (CW) in all thelodont scales pertaining to topological series that were classified into the morphotype 5 (drag reduction scales) after their inclusion in the CVA-1; and later, (2) we have carried out a correlation analysis between ARD and CW in each species for testing the existence of decoupling between these two variables. The measurements were taken with ImageJ software 1.46r and correlation analysis was performed using PASW Statistics 18 software.

## Results

### Squamation patterns in extant sharks

#### Morphometric characterization of placoid scales

Numerical values for measured variables in all shark scales are presented in S5 Table. CVA-1 generated seven discriminant functions whose eigenvalues, proportions of explained variance, canonical correlations and standardized coefficients are showed in Table 3. The first discriminant function explains 67.3% of the variance, whereas the second one explains 15.3% (82.6% of total variance). The significance of the discriminant functions tested hierarchically with Wilks’ lambda and the pairwise group comparisons provided validity for the canonical discriminant analysis to distinguish among the eight morphotypes (S6 Table). The graph of the first two discriminant functions shows good separation among the 8 morphotypes with a slight overlapping of morphotypes 7 (of abrasion protective function) and 8 (of generalized functions) (Fig 3A). The discriminant analysis correctly classified 96.4% (54 of 56 scales) of the original cases and 94.6% (53 of 56 scales) of the cross-validated cases (Table 4; the detailed results of original and cross-validated cases are shown in S6 Table).

**Table 3:** Summary statistics for Canonical Variate Analysis-1 (CVA-1, with eight scale morphotypes as defined groups and ten size-free variables of the crown surface as discriminant variables). Eigenvalues, proportions of explained variance, canonical correlations and standardized coefficients are shown for each discriminant function.

	Function 1	Function 2	Function 3	Function 4	Function 5	Function 6	Function 7
**Eigenvalue**	65.109	14.847	12.795	2.262	1.373	0.315	0.064
**% of variance**	67.285	15.343	13.223	2.338	1.419	0.326	0.066
**Cumulative %**	67.285	82.628	95.851	98.189	99.608	99.934	100.000
**Canonical correlation**	0.992	0.968	0.963	0.833	0.761	0.490	0.246
**Standardized coefficients**							
*Circularity*	0.705	0.037	-0.257	0.101	1.420	-0.367	0.443
*Solidity*	-0.275	-0.524	0.283	0.674	-1.021	0.698	0.185
*MLC/MWC*	-0.122	0.548	0.770	0.142	0.767	-0.007	0.328
*RLA/MLC*	0.468	0.752	-0.214	1.467	0.647	0.547	-0.643
*RLA*^*2*^*/ACS*	0.153	0.315	-0.549	-0.056	-0.460	-0.764	0.448
(MLC*MWC)/ACS	0.325	0.469	-0.116	0.050	0.448	0.809	0.626
*LLR/MLC*	0.517	-0.628	0.750	-1.287	-0.471	-0.211	0.407

**Table 4:** Summary results of the original and cross-validated classification for all scales of known morphotype and functional type in Canonical Variate Analysis-1 (CVA-1, with eight scale morphotypes as defined groups and ten size-free variables of the crown surface as discriminant variables).

		Predicted functional type and morphotype								Classified correctly		Misclassified	
		*Biolum*.			*Defens*.	*Hydrod*.	*Abras*.		*General*.				
		*M*. *1*	*M*. *2*	*M*. *3*	*M*. *4*	*M*. *5*	*M*. *6*	*M*. *7*	*M*. *8*	Count	%	Count	%
**Original cases**													
*Biolum*.	*M*. *1*	7	0	0	0	0	0	0	0	7	100.0	0	0.0
*Biolum*.	*M*. *2*	0	7	0	0	0	0	0	0	7	100.0	0	0.0
*Biolum*.	*M*. *3*	0	0	7	0	0	0	0	0	7	100.0	0	0.0
*Defens*.	*M*. *4*	0	0	0	7	0	0	0	0	7	100.0	0	0.0
*Hydrod*.	*M*. *5*	0	0	0	0	7	0	0	0	7	100.0	0	0.0
*Abras*.	*M*. *6*	0	0	0	0	0	7	0	0	7	100.0	0	0.0
*Abras*.	*M*. *7*	0	0	0	0	1	0	5	1	5	71.4	2	28.6
*General*.	*M*. *8*	0	0	0	0	0	0	0	7	7	100.0	0	0.0
**Cross-validated cases**													
*Biolum*.	*M*. *1*	7	0	0	0	0	0	0	0	7	100.0	0	0.0
*Biolum*.	*M*. *2*	0	6	1	0	0	0	0	0	6	85.7	1	14.3
*Biolum*.	*M*. *3*	0	0	7	0	0	0	0	0	7	100.0	0	0.0
*Defens*.	*M*. *4*	0	0	0	7	0	0	0	0	7	100.0	0	0.0
*Hydrod*.	*M*. *5*	0	0	0	0	7	0	0	0	7	100.0	0	0.0
*Abras*.	*M*. *6*	0	0	0	0	0	7	0	0	7	100.0	0	0.0
*Abras*.	*M*. *7*	0	0	0	0	1	0	5	1	5	71.4	2	28.6
*General*.	*M*. *8*	0	0	0	0	0	0	0	7	7	100.0	0	0.0

96.4% of original grouped cases correctly classified.

94.6% of cross-validated grouped cases correctly classified.

#### Specific squamation patterns

The inclusion of the morphological variants present in the whole squamation of studied specimens in the CVA-1 permitted their assignments to one morphotype and one concrete functional type (Fig 3B and S7 Table), allowing us to calculate the body surface covered by each scale morphotype and each functional type in all species (in terms of percentage of the whole body surface). S8 Table shows coverage percentages occupied on the whole body, as well as on the dorsal and the ventral sides of the body separately.

All studied specimens possess from one to three different scale functional types although most of them show a combination of only two. In that case, the specimens usually possess abrasion resistant scales (morphotype 6) covering small body areas, as snout, fin leading edges, surrounding mouth area and claspers, together with another functional type that covers most of body surface. Other functional type combinations are rare; only abrasion resistant scales (morphotypes 6 or 7) and scales with generalized functions (morphotype 8) occur together often with similar ratios in some specimens. Almost all studied specimens are entirely covered by scales with the exception of *Deania calcea*, *Squatina aculeata* and some bioluminescent sharks that have naked areas. In addition, our analysis confirms some common features of sharks squamations noted by Reif [15]: (1) rhomboidal smooth scales are found on the fin leading edges of most species, (2) scales of similar morphology but smaller size than those present on the body surface often occur around the gill openings, (3) smooth circular scales usually cover the claspers and nictitating membranes, (4) many species have modified scales around the free neuromasts and (5) smooth rounded scales are always present on the snout area.

### Relationship between squamation pattern and lifestyle in extant sharks

#### Squamation pattern and lifestyle; Quantitative approach

**Fig 4:**
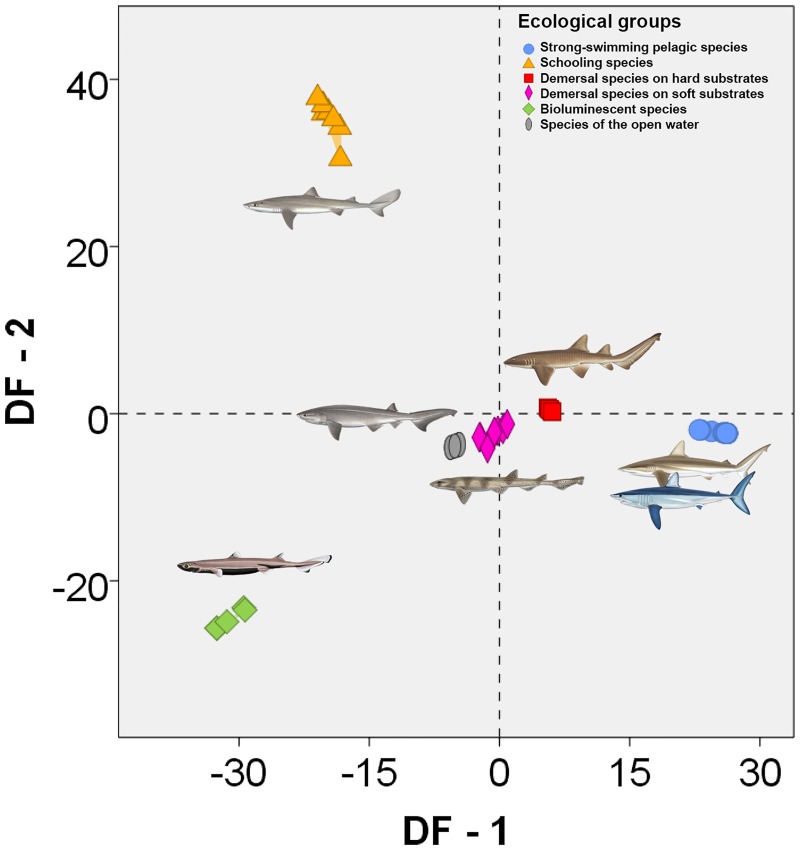
Canonical Variate Analysis-2 (CVA-2), six ecological groups of sharks taken as defined groups (modified from Reif [15]) and percentages of coverage of each scale morphotype used as discriminant variables. Results are plotted based on the first two discriminant functions. All shark drawings taken from Shark Trust [72], courtesy of Marc Dando.

The CVA-2 generated five discriminant functions whose eigenvalues, proportions of explained variance, canonical correlations and standardized coefficients are available in Table 5. The first discriminant function summarizes 49.3% of the total variance, while the second one summarizes 34.2% (83.5% of total variance). The graph of the first two discriminant functions shows good separation among all groups (Fig 4) and results of discriminant functions test and pairwise group comparisons suggest that the analysis is able to distinguish all ecological groups (S9 Table). The discriminant analysis correctly classified 100% (56 of 56 individuals) of the original cases and 98.2% (55 of 56 individuals) of the cross-validated cases (Table 6; the detailed results of original and cross-validated cases are shown in S9 Table). Furthermore, results of the two additional CVAs performed considering only the coverage percentages of each scale morphotype on the dorsal and ventral sides of the body respectively are shown in detail in S10 and S11 Tables.

**Table 5:** Summary statistics for Canonical Variate Analysis-2 (CVA-2, with six ecological groups as defined groups and scale morphotype percentages of coverage on the whole body as discriminant variables). Eigenvalues, proportions of explained variance, canonical correlations and standardized coefficients are shown for each discriminant function.

	Function 1	Function 2	Function 3	Function 4	Function 5
**Eigenvalue**	442.425	306.212	134.818	13.200	0.000
**% of variance**	49.342	34.150	15.036	1.472	0.000
**Cumulative %**	49.342	83.492	98.528	100.000	100.000
**Canonical correlation**	0.999	0.998	0.996	0.964	0.005
**Standardized coefficients**					
*Morphotype 4*	0.233	1.297	0.061	-0.029	0.001
*Morphotype 5*	1.757	0.697	0.025	-0.063	0.001
*Morphotype 6*	6.109	4.097	4.159	0.820	-0.345
*Morphotype 7*	6.755	4.685	4.794	0.493	0.678
*Morphotype 8*	2.392	1.922	1.928	-0.793	0.006

**Table 6:** Summary results of the original and cross-validated classification for all studied sharks in Canonical Variate Analysis-2 (CVA-2, with six ecological groups as defined groups and scale morphotype percentages of coverage on the whole body as discriminant variables).

	Predicted ecological group						Classified correctly		Misclassified	
	*Near-shore*	*Schooling*	*Hard substrate*	*Soft substrate*	*Bioluminescent*	*Open water*	Count	%	Count	%
**Original cases**										
*Strong-swimming*	16	0	0	0	0	0	16	100.0	0	0.0
*Schooling*	0	8	0	0	0	0	8	100.0	0	0.0
*Hard substrate*	0	0	8	0	0	0	8	100.0	0	0.0
*Soft substrate*	0	0	0	8	0	0	8	100.0	0	0.0
*Bioluminescent*	0	0	0	0	8	0	8	100.0	0	0.0
*Open water*	0	0	0	0	0	8	8	100.0	0	0.0
**Cross-validated cases**										
*Strong-swimming*	16	0	0	0	0	0	16	100.0	0	0.0
*Schooling*	0	8	0	0	0	0	8	100.0	0	0.0
*Hard substrate*	0	0	8	0	0	0	8	100.0	0	0.0
*Soft substrate*	0	0	1	7	0	0	7	87.5	1	12.5
*Bioluminescent*	0	0	0	0	8	0	8	100.0	0	0.0
*Open water*	0	0	0	0	0	8	8	100.0	0	0.0

100.0% of original grouped cases correctly classified.

98.2% of cross-validated grouped cases correctly classified.

#### Squamation pattern and lifestyle; Qualitative descriptions

**Fig 5:**
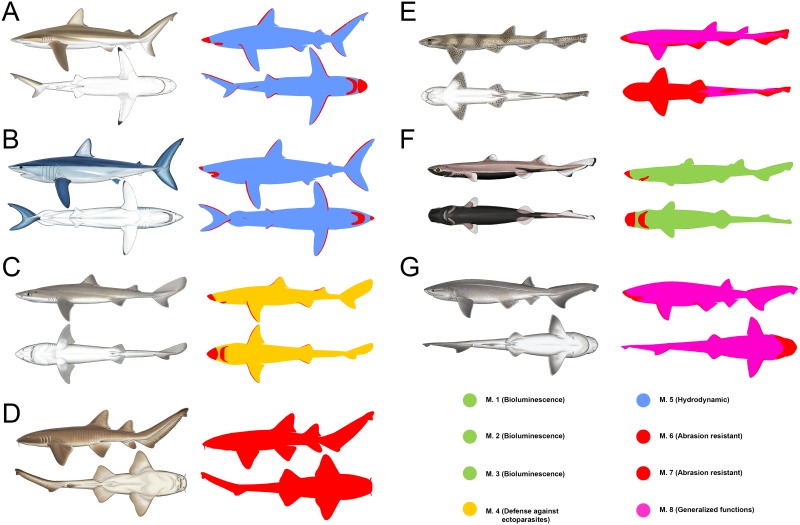
Characteristic squamation pattern of the seven ecological groups of sharks defined by Reif [15] showing the body distribution of scale morphotypes and functional types. (A) Large near-shore hunters (illustrated by *Carcharhinus brachyurus*). (B) Fast pelagic hunting species (illustrated by *Isurus oxyrinchus*). (C) Schooling species of low to moderate speed (illustrated by *Squalus acanthias*). (D) Demersal species on rocky substrates and in caves (illustrated by *Ginglymostoma cirratum*). (E) Demersal species on sandy and muddy substrates (illustrated by *Scyliorhinus canicula*). (F) Mesopelagic luminescent species (illustrated by *Etmopterus spinax*). (G) Slow species of the open water (illustrated by *Hexanchus griseus*). All shark drawings taken from Shark Trust [72], courtesy of Marc Dando. Scale drawings modified from Compagno [62, 63, 64].

The arrangement of analyzed shark taxa into ecological sets allowed us to evaluate some general characteristics of the squamation patterns for each ecological group (Fig 5):

1. Large near-shore hunters and fast pelagic hunting species (Fig 5A and 5B; strong-swimming pelagic species). Squamation patterns of these two ecological groups can be considered identical regarding the topological distribution of the scale functional types. In both cases the body is completely covered with drag reduction scales (morphotype 5) except for the snout which is covered with abrasion resistant scales (morphotype 6), sometimes restricted to a few rows as in hammerhead sharks.
2. Schooling species of low to moderate speed (Fig 5C). The body is mostly covered with defensive scales against ectoparasites and the settlement of epibionts (morphotype 4) although other functional types are present covering small areas in some species of the group (e.g. *Deania calcea* and *Galeorhinus galeus* juvenile).
3. Demersal species on rocky substrates and in caves (Fig 5D). The body of these sharks has a compact appearance, being completely covered with abrasion resistant scales (morphotypes 6 and/or 7). Usually there is no strong morphological differentiation in the scales of the snout, scales from the circum-oral region or scales of the fin leading edges.
4. Demersal species on sandy and muddy substrates (Fig 5E). The squamation pattern of this ecological group shows dorso-ventral differentiation. The dorsal side of the body is covered with scales of generalized functions (morphotype 8) whereas the ventral side is covered with abrasion resistant scales (morphotypes 6 or 7), mainly on the anterior half.
5. Mesopelagic luminescent species (Fig 5F). The body of these sharks is almost entirely covered with widely spaced scales that enable bioluminescence (morphotypes 1, 2 or 3) sometimes arranged in rows (e.g. *Etmopterus lucifer*). Photophores are located between scales and are visible under the binocular microscope as black dots, whose distribution and density varies between species (See [61]). Naked areas are usual in some parts of the body, on the leading edge of fins or, more commonly, on the trailing edge of fins.
6. Slow species of the open water (Fig 5G). The body is covered mostly with scales of generalized functions (morphotype 8) except for the snout and circum-oral region that are covered with abrasion resistant scales (morphotypes 6 or 7).

### Squamation patterns in thelodonts

#### Squamation patterns in thelodont species known from articulated squamations

Scales of articulated thelodonts were assigned to defensive scales against ectoparasites and the settlement of epibionts (morphotype 4), abrasion resistant function (morphotypes 6 and 7) and scales with generalized functions (morphotype 8) after their inclusion in the CVA-1 (S12 Table). Noticeably, none of them were assigned to bioluminescent scales (morphotypes 1, 2 and 3) or drag reduction scales (morphotype 5).

**Fig 6:**
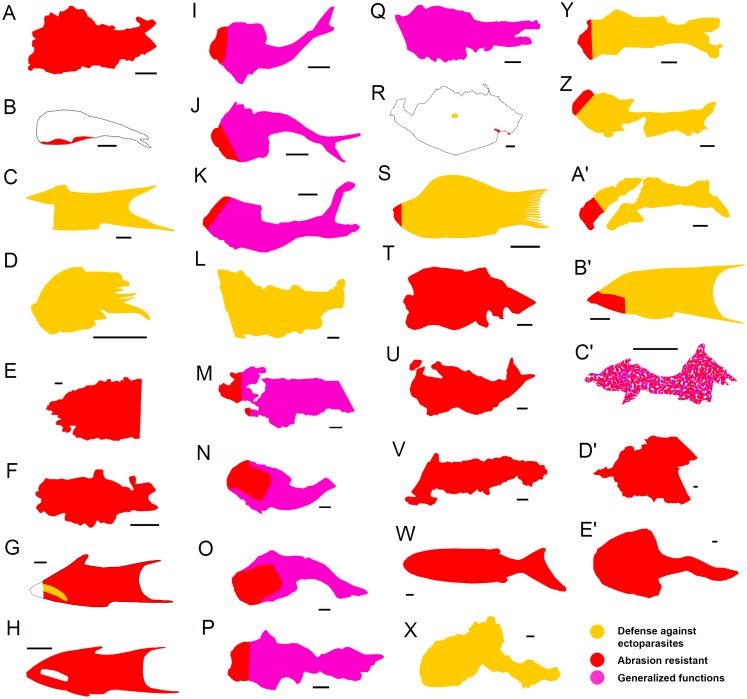
Distribution of scale functional types in articulated specimens of thelodonts, including complete and partial squamations and patches of scales. (A) *Archipelepis bifurcata* (GSC 117 187) (B) *Archipelepis turbinata* (UALVP 32990). (C) *Cometicercus talimaae* (UALVP 33207). (D) *Drepanolepis maerssae* (UALVP 32917). (E) *Eestilepis prominens* (GSC 117 198). (F) *Erepsilepis margaritifera* (UALVP 43115). (G) *Furcacauda fredholmae* (reconstruction from several articulated remains) (H). *Furcacauda heintzae* (reconstruction from several articulated remains). (I) *Lanarkia horrida* (NMS.G.1991.48.3A). (J) *Lanarkia horrida* (NMS.G.1991.48.3B). (K) *Lanarkia lanceolata*, (NMS.G.1991.48.6). (L) *Lanarkia spinulosa* (GSE 5977). (M) *Loganellia prolata* (GSC 117 178). (N) *Loganellia scotica* (AM.F.89433A). (O) *Loganellia scotica* (AM.F.89433B). (P) *Loganellia sulcata* (UALVP 43150). (Q) *Loganellia sulcata* (GSC 117 177). (R) *Nikolivia milesi* (BMNH.P.53902). (S) *Pezopallichthys ritchiei* (reconstruction from several articulated remains). (T) *Phillipsilepis cornuta* (UALVP 43123). (U) *Phillipsilepis crassa* (GSC 117 182). (V) *Phillipsilepis pusilla* (GSC 117 186). (W) *Phlebolepis elegans* (reconstruction from several articulated remains). (X) *Shielia gibba* (GSC 117 181). (Y) *Shielia parca* (GSC 117 179). (Z) *Shielia taiti* (NMS.G.1991.48.7A). (A’) *Shielia taiti* (NMS.G.1991.48.7B). (B’) *Sphenonectris turnerae* (reconstruction from several articulated remains). (C’) *Thelodus laevis* (TUG 1025–1052). (D’) *Thelodus macintoshi* (MCZ 2035). (E’) *Turinia pagei* (NMS.G.1891.92.133). Specimens of *Illoganellia colossea* (UALVP 43129, GSC 117 199) and *Thelodus inautditus* (UALVP 43141) are not figured because the morphology of their patches is not defined in the literature. Anterior is left for all specimens. Scale bars, 1cm.

The arrangement of each scale functional type in the studied specimens is shown in Fig 6. Percentages of the body area covered by each morphotype are available in S13 Table. CVAs-2.1–3 assigned all thelodont squamation patterns to three ecological groups: demersal species on rocky substrates and in caves, schooling species of low to moderate speed or slow species of the open water (Table 7 and S14 Table for summarized and detailed results respectively). The only exception is the species *Loganellia scotica*, which is assigned to demersal species on sandy and muddy substrates by CVA-2.1 but to slow species of the open water by CVA-2.3. This case is discussed in detail below. Note that the articulated specimen of *Thelodus laevis* could not be included in any of the CVAs because of the presence of different morphotypes interspersed together thus making it impossible to quantify their coverage areas.

**Table 7:** Summary results of the classification for all articulated thelodonts after their inclusion in Canonical Variate Analyzes-2.1–3 (CVA-2.1–3), with six ecological groups as defined groups and scale morphotype percentages of coverage on the whole body, ventral side or dorsal side of the body as discriminant variables respectively.

Species	Degree of preservation	Position of preservation	Registration number	ECOLOGICAL GROUP (CVA-2.1: Whole body)	ECOLOGICAL GROUP (CVA-2.2: Dorsal side of the body)	ECOLOGICAL GROUP (CVA-2.3: Ventral side of the body)
*Loganellia scotica*	Nearly complete squamation	Dorsal	AM.F.89433A	X	Soft substrate^1^	X
*Loganellia scotica*	Complete squamation	Ventral	AM.F.89433B	X	X	Open water
*Loganellia scotica*	Complete squamation	Dorsal + Ventral	AM.F.89433A + AM.F.89433B	Soft substrate	X	X
*Loganellia sulcata*	Complete squamation	Dorsal	UALVP 43150	X	Open water^2^	X
*Loganellia sulcata*	Nearly complete squamation	Probably Ventral	GSC 117 177	X	X	Open water
*Shielia taiti*	Complete squamation	Dorsal	NMS.G.1991.48.7A	X	Schooling	X
*Shielia taiti*	Complete squamation	Ventral	NMS.G.1991.48.7B	X	X	Schooling
*Shielia taiti*	Complete squamation	Dorsal + Ventral	NMS.G.1991.48.7A + NMS.G.1991.48.7B	Schooling	X	X
*Shielia gibba*	Complete squamation	Dorsal or Ventral	GSC 117 181	X	Schooling	Schooling
*Shielia parca*	Complete squamation	Dorsal	GSC 117 179	X	Schooling	X
*Phlebolepis elegans*	Reconstruction	Lateral	Pi-6685, 7050, 6686, 6682, 6731, 6728, *inter alia*	Hard substrate	X	X
*Erepsilepis margaritifera*	Complete squamation	Dorsal or Ventral	UALVP 43115	X	Hard substrate	Hard substrate^2^
*Lanarkia horrida*	Complete squamation	Dorsal	NMS.G.1991.48.3A	X	Open water^2^	X
*Lanarkia horrida*	Complete squamation	Ventral	NMS.G.1991.48.3B	X	X	Open water
*Lanarkia horrida*	Complete squamation	Dorsal + Ventral	NMS.G.1991.48.3A + NMS.G.1991.48.3B	Open water	X	X
*Lanarkia lanceolata*	Complete squamation	Dorsal or Ventral	NMS.G.1991.48.6	X	Open water^2^	Open water
*Phillipsilepis crassa*	Nearly complete squamation	Probably Ventral	GSC 117 182	X	X	Hard substrate^2^
*Phillipsilepis pusilla*	Nearly complete squamation	Dorsal or Ventral	GSC 117 186	X	Hard substrate	Hard substrate^2^
*Turinia pagei*	Complete squamation	Ventral	NMS.G.1891.92.133	X	X	Hard substrate^2^
*Furcacauda heintzae*	Reconstruction	Lateral	NMC 13753; UALVP 32462, 32947,32953, 32958, 32966	Hard substrate	X	X
*Furcacauda fredholmae*	Reconstruction	Lateral	UALVP 33024, 23154, 32417, 32949, 38074, 39085, 32462; NMC 13752, 13754	Hard substrate	X	X
*Cometicercus talimaae*	Nearly complete squamation	Lateral	UALVP 33207	Schooling	X	X
*Drepanolepis maerssae*	Complete squamation	Lateral	UALVP 32917	Schooling	X	X
*Sphenonectris turnerae*	Reconstruction	Lateral	UALVP 33023, 38075, 37149, 32920, *inter alia*	Schooling	X	X
*Pezopallichthys ritchiei*	Reconstruction	Lateral	UALVP 32994, 29922, 29925, 32991, 32992, 32993, 32995, 32997, 33001, 33002, 33004, 33005, 33007–33009	Schooling	X	X

Non conclusive assignments:

^1^Specimen that could pertain also to slow species of the open water;

^2^Specimens that could also pertain to demersal species on soft substrates (see text).

#### Squamation patterns in thelodonts known from disarticulated remains

As scales of articulated thelodonts, the scales of each topological series of thelodonts known from disarticulated remains were assigned to scale morphotypes after their inclusion in the CVA-1 (Fig 3C and S15 Table). Posterior probabilities (probability of belonging to the group) are high, close to 1 in most cases, which indicates that the assignments of thelodont scales into scale morphotypes were done reliably. Mahalanobis distance to the centroid and conditional probabilities (probability of obtaining a discriminant punctuation as obtained or more extreme within the group) of thelodont scales are similar to those of shark scales for all morphotypes except for morphotypes 4 and 8, which show slightly higher Mahalanobis distance and lower values of conditional probabilities (Fig 3D and compare also S6, S7, S12 and S15 Tables).

CVA-1 assigned the scales of the 124 species of thelodonts know from disarticulated remains mostly to defensive scales against ectoparasites and the settlement of epibionts (morphotype 4), abrasion resistant scales (morphotypes 6 and 7), and scales with generalized functions (morphotype 8) (S15 Table). Abrasion resistant scales are present in 111 species (90%) as a unique functional type or in combination with others whereas defensive scales and scales with generalized functions are present in 38 (31%) and 21 (17%) species respectively, usually in combination with abrasion resistant scales. In addition, drag reduction scales (morphotype 5) have been found in ten species (9%) belonging to at least five different families (Shieliidae, Katoporididae, Thelodontididae, Apalolepididae and Furcacaudidae). Only scales of the species *Longodus acicularis* were assigned to bioluminescent functional types (morphotypes 2 and 3) although this assignment is discussed in detail in the Discussion section. In general, head and transitional scales (sensu series of Gross [42]; see above) have been classified here as scales of abrasion protective function, whereas trunk scales display wider diversity in functional types.

In most cases, each topological series corresponds to a unique well-defined scale morphotype. However, in 16 species some series present scales that belong to more than one morphology corresponding to different functional types. This variability usually occurs within trunk scale series with the only exception being the species *Nikolivia aligera* (Nikoliviidae) where both scales for protection against abrasion and scales for defense against ectoparasites and the settlement of epibionts are found within head series scales. On the other hand, two combinations of functional types are found in trunk scales of the remaining species: defensive scales against ectoparasites and the settlement of epibionts together with abrasion resistant scales in five species and scales with generalized functions together with abrasion resistant scales in ten species. The presence of two different functional types within a same topological series does not allow for the estimation of their body coverage area, which impedes the inclusion of these species in the second discriminant analysis (CVA-2.1). The same occurs in species known only from a few number of scales which lack representation of some topological series (usually head and/or transitional series for being the less abundant in relative terms). In sum, from the 124 species known by disarticulated remains, 77 were incorporated in the CVA-2.1. The estimated body coverage percentages for each morphotype and functional type and the assignments within ecological groups for all species are shown in S16 Table. Most of the species were assigned to demersal species on rocky substrates and in caves (40 spp., 52%) and to schooling species of low to moderate speed (22 spp., 29%). The rest of species were assigned to slow species of the open water (8 spp., 10%) and strong-swimming pelagic species (6 spp., 8%). One thelodont species, *Longodus acicularis*, was assigned to mesopelagic luminescent species by the analysis (but this assignment is discussed below) and none to demersal species on sandy and muddy substrates.

The remaining 47 species known from disarticulated scales could not be included in the CVA-2.1. Nevertheless, some aspects of the squamation pattern can be inferred qualitatively allowing us to include a number of these species into one ecological group (S15 Table and see also S1 Appendix). The previous analysis on sharks evidences that the presence of certain scale morphotypes (or combinations) can be linked specifically with one ecological group (see below). According to this, species with defensive scales against ectoparasites and epibionts (morphotype 4) were included into schooling species and species with drag reduction scales (morphotype 5) where included into strong-swimming pelagic species, as both morphotypes are restricted to these ecological groups respectively. In the same sense, species with only abrasion-resistant scales within the trunk series were included into demersal species on rocky substrates and in caves whereas species with only scales of generalized functions within the trunk series were assigned to slow species of the open water. Finally, the co-occurrence of abrasion-resistant scales and scales of generalized functions within the trunk region is exclusive of demersal sharks that inhabit on sandy and muddy substrates. In consequence, thelodonts that show this combination were assigned to this ecological group (but see below).

Results of the additional morphometric analysis performed for thelodont species with drag reduction scales (morphotype 5) are shown in Table 8. All calculated average ridge distances (ARD) are comprised between 35.8 and 83.5 μm, varying over a narrow range of values within the same species (standard deviations from 5.1 to 12.1 μm). Generally, crown width (CW) means range between 200 and 500 μm, although scales of two species clearly exceed these measures (*Apalolepis obruchevi* with 708.0 μm and *Apalolepis angelica* with 1086.3 μm). The variability in CW differs among species, some thelodonts display a remarkable uniformity in CW (e.g. *Canonia kaerberi*, CW SD and CW CV equal to 25.2 μm and 11.0% respectively) whereas others show wider variation (e.g. *Thelodus visvaldi*, CW SD and CW CV equal to 185.1 μm and 44.0% respectively). Finally, no significant correlation between ARD and CW has been found in any of the ten studied species.

**Table 8:** Means, standard deviations and coefficient of variation of Crown Width (CW) and Average Ridge Distance (ARD) in drag reduction scales of ten different species of thelodonts. For comparison, the same data have been compiled for 14 species of extant sharks belonging to the two different ecological groups of strong-swimming pelagic sharks (fast pelagic hunting species and large near-shore hunters following the ecological classification of Reif [15]). Correlation analysis results between CW and ARD are given for each species of thelodonts.

Species	Source	ARD mean	ARD SD	CW mean	CW SD	CW CV	Correlation results	
							Pearson coefficient	Sig.
**THELODONTS WITH DRAG REDUCTION SCALES**								
*Praetrilogania grabion*	[78]	45.8	9.7	215.3	50.3	23.4%	0.494	0.176
*Trimerolepis gemella*	[57]	60.5	12.1	289.2	64.0	22.1%	-0.223	0.375
*Thelodus visvaldi*	[79]	75.5	9.7	421.1	185.1	44.0%	0.272	0.602
*Apalolepis obruchevi*	[43]	83.5	7.5	700.8	91.2	13.0%	-0.579	0.607
*Apalolepis angelica*	[80]	38.0	8.0	1086.3	162.8	15.0%	0.967	0.163
*Apalolepis brotzeni*	[43]	40.8	5.3	508.6	67.1	13.2%	-0.114	0.855
*Skamolepis fragilis*	[43]	48.0	7.0	352.0	43.3	12.3%	-0.942	0.217
*Canonia grossi*	[3]	41.4	9.6	259.9	71.7	27.6%	0.272	0.419
*Canonia costulata*	[57]	49.4	11.9	235.5	59.4	25.2%	0.439	0.089
*Canonia kaerberi*	[81]	35.8	5.1	228.2	25.2	11.0%	-0.252	0.683
**SHARKS (Fast pelagic hunting species)**								
*Isurus oxyrinchus*(220 cm)	[15]	40.9	3.8	147.7	32.8	22.2%		
*Lamna nasus* (130 cm)	[15]	67.7	5.6	284.5	23.0	8.1%		
*Carcharhinus falciformis* (37 cm)	[15]	58.3	9.4	177.1	19.6	11.1%		
*Carcharhinus falciformis* (227 cm)	[15]	62.1	4.9	365.5	66.0	18.1%		
*Carcharhinus galapagensis* (225 cm)	[15]	82.4	10.2	455.6	65.7	14.4%		
*Sphyrna tudes* (26 cm)	[15]	38.6	3.9	135.5	10.6	7.8%		
*Sphyrna tudes* (120 cm)	[15]	41.3	3.8	212.0	16.6	7.8%		
Mean		**55.9**	**5.9**	**254.0**	**33.5**	**13.2%**		
**SHARKS (Large near-shore hunters)**								
*Carcharhinus melanopterus* (40 cm)	[15]	66.4	8.7	256.4	36.3	14.2%		
*Carcharhinus melanopterus* (137 cm)	[15]	102.2	27.4	562.2	139.0	24.7%		
*Carcharhinus amblyrhynchos* (158 cm)	[15]	91.2	15.8	561.2	120.7	21.5%		
*Carcharhinus milberti* (39 cm)	[15]	63.3	7.3	221.3	27.2	12.3%		
*Carcharhinus milberti* (169 cm)	[15]	100.9	18.6	460.9	123.1	26.7%		
*Prionace glauca* (42 cm)	[15]	93.1	16.0	231.6	75.8	32.7%		
*Prionace glauca* (234 cm)	[15]	102.0	29.9	217.5	66.5	30.6%		
Mean		**88.4**	**17.7**	**358.7**	**84.1**	**23.4%**		

## Discussion

### Squamation patterns and ecology in sharks

**Fig 7:**
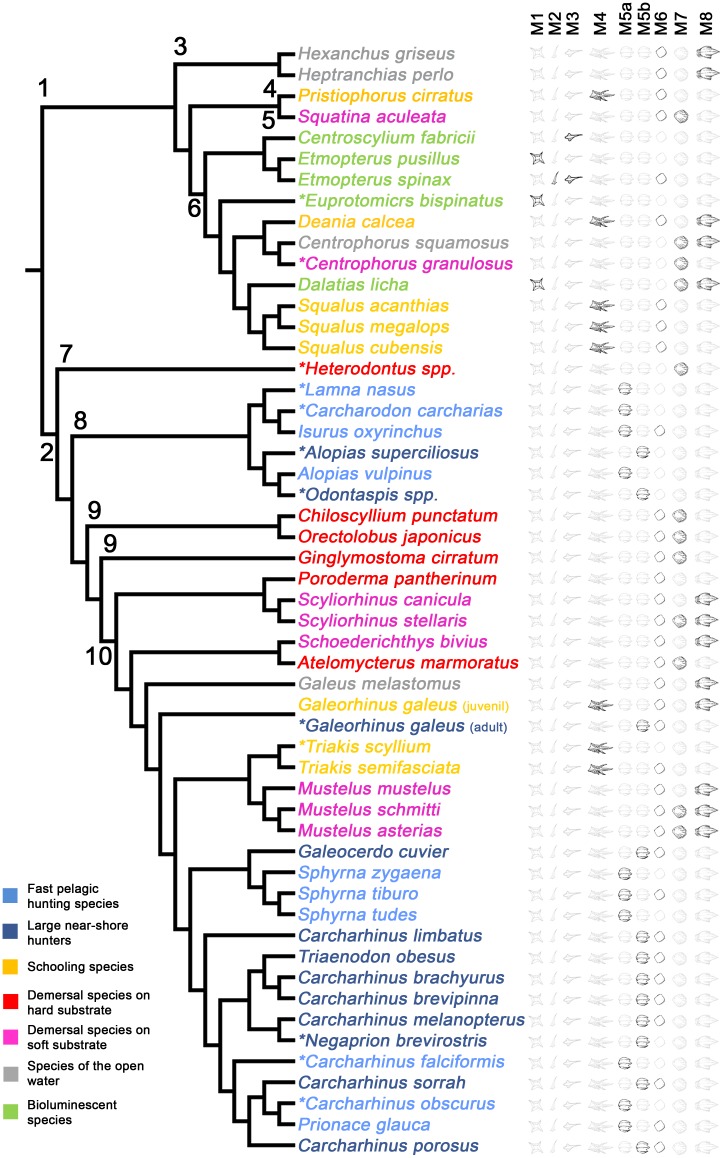
Phylogeny of sharks, with distribution of ecological groups and scale morphotypes. More than 50 species are represented, including taxa studied here and some taxa examined by Reif [15] (denoted with asterisk). M5 is separated in two morhotypes (M5a and M5b) according to the distance betwen ridges (see Table 1 and text). Colours specify ecological assignation and numbers indicate major phylogenetic groups (1, Squalomorphii; 2, Galeomorphii; 3, Hexanchiformes; 4, Pristiophoriformes; 5, Squatiniformes; 6, Squaliformes; 7, Heterodontiformes; 8, Lamniformes; 9, Orectolobiformes; 10, Carcharhiniformes). *Galeorhinus galeus* is denoted with two different colors as the ecology of juveniles is different than that of the adults. Phylogenetic interrelationships are based on Vélez-Zuazo and Agnarsson [85].

The squamation patterns of 53 species of extant sharks, whose ecology and life habits are well known, have been studied here with the aim of establishing a comparative framework useful for inferring some aspects of the ecology of thelodonts. The studied species were included in one of the seven ecological groups differentiated by Reif [15] on the basis on several general aspects of their ecology and habitat preferences. Obviously, the wide ecological diversity of the group lets to the establishment of more detailed classifications (see for example the arrangement of chondrichthyans into 18 ecomorphotypes proposed by Compagno [82]). However, groups proposed by Reif [15] are simple enough to approach the study of fossil taxa and infer some general aspects of their lifestyle, thus being suitable for the present work. In fact, our analysis show that this classification is well founded and the species of each group share a set of well-defined ecological parameters (Fig 2). It is worth discussing here that as a result of the phylogenetic legacy closely related species tend to exhibit both morphological and ecological similarities. Such similarity could be on occasions the result of shared ancestry instead of an indicator of the correlation between the morphology and ecology (e.g, [83, 84]). However, this possibility is quite improbable in our case, as the independent evolution of the same ecology and scale morphotypes in non-closely related sharks has been evidenced by several authors [13, 14, 15] suggesting a low phylogenetic signal in this sense. In fact, when ecological assignment obtained for the taxa studied here are considered in a phylogetical context (Fig 7), it revelas a high degree of convergence, with most of the ecologogical goups evolving in distant lineages multiple times. For instance, three of the ecological groups considered (demersal species living on soft substrates, species of the open water and schooling species) appear separately within the two major groups of sharks, Galeomorphii and Squalomorphii. Similarly, representatives of demersal species living on hard substrates are found in a number of species of three different orders of galean sharks (Orectolobiformes, Heterodontiformes and Carcharhiniformes). Strong-swimming pelagic species (fast pelagic hunting species and large near-shore hunters) are concentrated in two lineages, but they appear distanly nested in the phylogeny (including representatives in a terminal clade of carcharhiniforms and lamniforms). Finally, within squaliforms, which concentrate all bioluminescent sharks, these appear represented in two separate families (Etmopteridae and Dalatiidae) interspersed with other ecological strategies. Additional support for the fact that ecological pattern is not amplified by phylogeneny is provided by the existence of ontogenetic changes in the squamation of species that undergo ecological shifts throughout their life (e.g., in *Galeorhinus galeus*) and the lack of such phenomenon in species where juveniles and adults show similar lifestyles.

Scale morphologies and functional types described by Reif [14, 15] (see above) have been characterized from metric variables that are easily measurable in isolated scales of sharks. In consequence, the same measurements can be used for the study of the squamation in thelodonts, which are largely known only from assemblages of disarticulated scales. Some of these variables have already been successfully used in previous works for distinguishing or characterizing placoid scale morphologies by classical morphometrics [13, 17, 19, 20, 69, 86]. Regarding Canonical Variate Analysis (CVA-1), the representation of discriminant punctuations of original cases (Fig 3A) reveals a clear separation between all morphotypes. Dispersion increases noticeably for all groups when shark scales of unknown morphotype are included in the analysis however the different morphotypes are still recognized as well-separated natural groups (Fig 3B). A slight overlapping occurs only between morphotypes 7 (abrasion resistant scales) and 8 (scales with generalized functions), which could be expected since the morphology of morphotype 8 is probably the result of a trade-off between more than one function. The CVA-1correctly classified nearly 95% of the cross-validated cases into their correct morphotype (Table 4), demonstrating a high predictive power. It provides promise for inferring function in micromeric scales, which is indeed a first critical step to understand the relationship between squamation and lifestyle.

In his atlas of squamation and ecology of sharks, Reif [15] assigned one scale functional type for each ecological group although he noted the morphological variation present in a given individual. In fact, when morphometric analysis is applied to the whole of studied sharks, the result evidences that up to three different scale morphotypes, belonging to up to three functional types, can be present in the same specimen (S7 and S8 Tables). Indeed, only some morphotypes can be linked specifically with one ecological group. Scales associated with hydrodynamic function are present only in strong-swimming pelagic species. This functional type is by far the best studied to date given its possible biomimetic applications for enhancing the swimming performance in submerged bodies (see [87] and references therein). The role of scales with pronounced parallel riblets in drag reduction has been demonstrated many times, due to their passive flow control by training the vortices in the direction of flow, reducing overall skin friction. (e.g. [19, 87, 88, 89, 90]). Reif [15] distinguished two characteristic morphologies for this functional type (Table 1) and, in relation to them, recognized two separate ecological groups: fast pelagic hunting sharks and large near-shore hunters (unified into strong-swimming pelagic species in the present study). In the scales of the former, the distance between ridges comprises between 35 and 80 μm, ranging within narrow values for each species independently of the variability on scale crown width (Table 8). Two different strategies have evolved for maintaining this distance comparatively constant: (1) some sharks reduce the variability in scale width and keep constant the number of ridges (e.g. *Lamna nasus*, smallest *Carcharhinus falciformis* and both *Sphyrna tudes* specimens in Table 8) whereas others (2) decouple these two variables adding further ridges in wider scales (e.g. biggest *Carcharhinus falciformis*, *Isurus oxyrinchus* and *Carcharhinus galapagensis* specimens in Table 8). In the scales of large near-shore hunters the distance between ridges is usually wider than 80 μm. and is not independent of crown width, thus existing a strong positive correlation between both variables [15, 16]. Scales related to bioluminescence (morphotypes 1, 2 and 3) are exclusive of mesopelagic luminescent species covering an important percentage of their body surface. Both the shape and insertion angle of the crown and the low density of coverage support their involvement in bioluminescent function, enabling the accommodation of photophores and the passage of the light emitted by them [15, 61]. Nevertheless the morphological disparity present in this functional type (three different morphotypes) suggests that these scales could be involved in other specific functions. Finally, scales of morphotype 4 are exclusive of schooling species of low to moderate speed. Due to the spine-like and the upward direction of their crowns, some authors have proposed defensive functions against ectoparasites and the settlement of epibionts for them [14, 15, 20]. However, although further investigation is needed before the full extent of this relationship can be evaluated, this study clearly evidences that there is a link between scales of morphotype 4 and the presence of schooling or shoaling in sharks. On the other hand, as said above, according with Reif [15] scales specialized in a defensive function against ectoparasites and the settlement of epibionts cover also part of the body of demersal sharks inhabiting muddy or sandy substrates and of the slow species of the open water. Nevertheless our morphometric analysis included these cases into scales of generalized functions (morphotype 8; Fig 5E–5G and S8 Table). Slow species of the open water include non-bioluminescent sharks that inhabit deep waters on the continental slope. These sharks show a benthopelagic lifestyle, swimming slowly both near the bottom and in the midwater, without lying in constant contact with the substrate. However, due to difficulty of conducting direct studies on deep water fishes many other aspects about their biology and their behavior remain unknown. Up to date schooling behavior has not been reported in any of the studied species included in this group, and only the occurrence of groups in *Hexanchus griseus* has been mentioned by Compagno [73] and Ebert et al. [75]. In consequence, given the lack of strong selective pressures related to drag force, abrasion, parasitism and bioluminescence, it is expected that the scales of most slow sharks of the open water are not clearly specialized in any of these functions. The same can be argued for scales of the dorsal body side of sharks inhabiting muddy or sandy substrates where the main abrasive stress occurs in the ventral part and most of them are sedentary sharks that rarely aggregate. Anyway, the precise function of these scales remains to be fully evaluated and will probably require much more research effort.

In contrast, the presence of all other remaining morphotypes cannot be linked with any clearly defined ecological group. For example, abrasion resistant scales (morphotypes 6 or 7) are present in all the ecological groups. In strong-swimming pelagic species (fast pelagic hunting species and large near-shore hunters), schooling species of low to moderate speed, mesopelagic luminescent species and slow species of the open water, the scales of this functional type are located almost exclusively on the snout and the circum-oral region, occupying a very low percentage of body surface rarely exceeding the 15% (Fig 5A–5C, 5F and 5G and S8 Table). These two areas are usually subjected to strong interaction with prey and/or the substrate during feeding. In demersal sharks, which spend most of their time moving in contact with the substrate, the percentage of body surface covered with scales of morphotypes 6 and 7 is higher, ranging between approximately 30% and 100% (S8 Table). In the case of demersal species on sandy and muddy substrates these scales are usually restricted to the ventral part of the body (Fig 5E) whereas in demersal species on rocky substrates and in caves they may cover the entire body (Fig 5D). In any case, scales with morphotypes 6 and 7 appear always located in areas subjected to abrasion stress. This fact together with their considerable thickness and the usual presence of scratch marks on the crown surface, evidence their involvement in abrasion strength function. Interestingly, scales of the fin leading edges as well as rounded scales of the claspers (both present in the majority of studied species) are classified into morphotype 6 by CVA-1. The role of these scales in protection against abrasion seems evident, although their distinctive morphologies suggest a compromise with other functions. In this sense, Reif [15] related rhomboidal scales of the leading edges to hydrodynamic aspects, at least for strictly pelagic species, conferring rigidity to the fin and those of the claspers to friction reduction. Scales of generalized functions (morphotype 8) are probably the result of a trade-off between more than one function or the absence of strong selective pressures of ectoparastism, bioluminescence, abrasion or hydrodynamics. They cover significant areas in demersal species on sandy and muddy substrates and in slow species of the open water (S8 Table). In the former, scales of this functional type are situated in body regions subjected to low abrasive stress (i.e., the dorsal side of the body; Fig 5E). In species of the open water, scales of generalized functions cover most of the body surface including the ventral side area (Fig 5G), probably subjected to less abrasive stress than in demersal species on sandy and muddy substrates as a consequence of a more bentohopelagic lifestyle. Scales of this functional type can also be present in small proportions in other ecological groups such as schooling species of low to moderate speed (e.g. in *Deania calcea* or in juvenile specimen of *Galeorhinus galeus*) and mesopelagic luminescent species (e.g. *Dalatias licha*) (S8 Table). However, scales assigned to generalized functions in these species have very low values of conditional probabilities, this meaning that their morphologies constitute a rare case within the usual morphological variability of this functional type (S7 Table). In fact, in these cases, scales assigned to generalized functions cover only very small areas restricted to an intermediate position between the abrasion resistant scales of the snout and the main scale functional type of the trunk, suggesting that they could be only transitional morphologies.

In consequence, the presence of several scale morphotypes (and functional types) in each ecological group makes necessary the quantification of this variability (in terms of percentage of body area covered by each morphotype) in order to obtain a better understanding of the relationship between squamation and the ecology in sharks. As expected, a second Canonical Variate Analysis (CVA-2), taking as defined groups the six ecological groups and including as discriminant variables the coverage percentages of each scale morphotype, confirms the close connection between squamation pattern and lifestyle in sharks. Results of discriminant function test and pairwise group comparisons (S9 Table) indicate that the analysis is able to distinguish between all ecological groups and has an excellent predictive power, classifying properly 98.2% of the cross-validated cases (Table 6 and S9 Table). In fact, all the specimens are classified rightly by CVA-2 with high posterior and conditional probability values (S9 Table) with the only exception of *Squatina aculeata* (MCTA-00092). Spiny Angelshark (*S*. *aculeata*) inhabits near the seabed of the outer continental shelf and uppermost slope (depths of 30 to 500 m.) on muddy bottoms [76], thus fitting well into the ecological group of demersal species on sandy and muddy substrates. However CVA-2 assigned it to demersal species on rocky substrates and in caves. Although Spiny Angelshark shows a clear morphological difference between scales of the ventral body side (with circular smooth crowns) and dorsal body side (with prominent ridges and upward directed crowns), CVA-1 classifies both morphological variants as scales of abrasion protective function (morphotypes 6 and 7 respectively; S7 Table). However, scales of the dorsal side, that were assigned to morphotype 7, show low values of conditional and posterior probabilities (0.003 and 0.512 respectively) and have been assigned to morphotype 8 (generalized functions) as the second highest group with similar posterior probabilities (0.445). This, together with the fact that demersal species on rocky substrates and in caves do not show a so obvious dorso-ventral differentiation, indicates that squamation pattern of Spiny Angelshark is in fact more similar to that present in demersal species on sandy and muddy substrates. This distinctive morphological differentiation has been previously noted by other authors who suggested that scales of dorsal side may fulfill defensive functions [15, 20].

Furthermore, our analysis is able to relate ontogenetic changes in the squamation patterns with the existence of different lifestyles in juveniles and adults of the same species. Squamation pattern of juveniles of *Galeorhinus galeus*, that remain in shallow estuaries or bays forming schools during the first two years of life [63, 73, 76], is assigned to schooling species of low to moderate speed (S9 Table); whereas adults, that can also be gregarious but show more pelagic habits, have the typical squamation of large near-shore hunters (see [15]). In contrast, juveniles of other species where no significant ontogenetic changes in lifestyle have been documented (e.g. *Sphyrna tiburo* and *Chiloscyllium punctatum*) are classified in the same ecological group than adults (S9 Table).

In conclusion, the good results obtained for sharks have allowed us to establish a reliable comparative framework useful for inferring lifestyles in thelodonts. Moreover, we propose that the described methodology could be applied for making ecological inferences in a significant number of groups with similar micromeric squamations, including both fossil groups without close extant representatives (e.g. astraspids, some heterostraceans, thelodonts, elegestolepids, mongolepids, acanthodians and most groups of elasmobranchs) as well as extant neoselachian species whose biology is still poorly known (e.g. deep-sea species).

Additionally, for the study of those thelodont species known only from squamations in dorsal or ventral view we have performed two additional Canonical Variate Analyses taking into account only the dorsal or ventral part of the body of sharks (CVA-2.2 and CVA-2.3 respectively). Cross-validation results suggest that CVA-2.2 is able to differentiate properly between individuals of all ecological groups except between demersal species on sandy and muddy substrates and slow species of the open water (S10 Table). In fact, both groups show a highly similar squamation on the dorsal body side, being covered mainly with scales of generalized functions (morphotype 8) whereas differences remain in the ventral body side (compare Fig 5G). The lack of the information relative to the squamation of the ventral side makes them indistinguishable from each other. In a similar way, cross-validated results show that CVA-2.3 differentiates properly between individuals of all ecological groups except between demersal species on sandy and muddy substrates and demersal species on rocky substrates and in caves (S11 Table). In this case, both groups show a highly similar squamation on the ventral body side, being covered mainly with scales of morphotype 6 and/or 7, whilst differences remain in the dorsal body side (compare Fig 5E). Consequently, the lack of the information relative to the squamation of the dorsal side makes them indistinguishable from each other. Therefore, these two aspects had to be taken into account for the correct interpretation of the results after the inclusion of thelodont squamations in both analyzes.

### Squamation patterns and ecology in thelodonts

The morphometric analysis performed here shows that the morphological diversity of thelodont scales fits well within the scale morphotypes and functional types established in living sharks (Fig 3D). Dispersion of some scale morphotypes in CVA-1 is slightly higher in thelodonts than in sharks although this could be the result of a bigger sample size (405 thelodont scales in comparison with 221 shark scales). The inclusion of a large number of new scales increases the probability of including rare cases that contribute to a higher dispersion in the point cloud (note that dispersion also increased noticeably when a higher number of shark scales was considered; Fig 3B). Anyway, our results suggest the presence of a higher morphological diversity, at least in some morphotypes, for thelodont scales and therefore their involvement in other more specific functions cannot be discarded (see below).

As previously indicated, several authors [3, 26] have noticed the similarities between the squamations of sharks and thelodonts regarding the patterns of scale morphological variation along the body. The analysis of the 29 thelodont species known from articulated remains provide statistical support for this, showing that topology and coverage areas of each scale functional type are also comparable in both groups (Figs 5 and 6). The results of CVA-2.1–3 (Table 7; see also S14 Table for detailed information) and a subsequent interpretation of the data (taken into account also the topological distribution of morphotypes and the presence of ecological group-specific functional types) allow recognizing three characteristic squamation patterns in articulated thelodonts clearly equivalent with some of those present in extant sharks:

1 Squamation patterns where the body is covered essentially by abrasion resistant scales (morphotype 6 or 7). This squamation pattern is characteristic of demersal sharks that inhabit on hard substrates such us rocky substrates, caves and reefs (Fig 5D). In thelodonts, this type of squamation has been recognized with reliability by CVA-2.1 in several species with complete specimens preserving both the dorsal and ventral squamations (*Furcacauda fredholmae*, *Furcacauda heintzae* and *Phlebolepis elegans*; Fig 6G, 6H and 6W and Table 7). In addition, *Eestilepis prominens*, know from a partial squamation ([57]: Fig 59) has also been reliably assigned qualitatively to this ecological group due to the only presence of abrasion resistant scales in both dorsal and ventral sides of the trunk (Fig 6E). The assignment of several other species with complete or partial articulated specimens preserved in ventral or indeterminate view (*Archipelepis bifurcata*, *Archipelepis turbinata*, *Erepsilepis margaritifera*, *Phillipsilepis cornuta*, *Phillipsilepis crassa*, *Phillipsilepis pusilla*, *Thelodus macintoshi* and *Turinia pagei*; Fig 6A, 6B, 6F, 6T, 6U, 6V, 6D’ and 6E’) was not conclusive; neither quantitatively, because the CVA-2.3 (considering only the squamation of the ventral area of the body) does not properly differentiate between demersal species on hard substrates and demersal species on soft substrates (Table 7), nor qualitatively, because of the high similarity between the ventral squamation patterns of both groups (compare Fig 5D and 5E). Notwithstanding this, *Archipelepis turbinata* and *Turinia pagei*, known from both articulated specimens and a large collection of isolated scales, are reliable assigned to demersal species on rocky substrates and in caves when disarticulated remains are taken into account (S16 Table and see also S1 Appendix). Finally, specimens of *Illoganellia colossea* and *Thelodus inauditus* are small patches of unknown body position where all scales are also assigned to morphotypes 6 or 7 (abrasion resistant scales). However, although these scale patches could fit well within the typical squamation pattern of demersal species of hard substrates, any other ecological group cannot be discarded for these thelodont species as abrasion resistant scales are found in small specific areas of all sharks.
2 Squamation patterns where most of the body is covered with scales specialized in a defensive function against ectoparasites and the settlement of epibionts (morphotype 4) and abrasion resistant scales are restricted to small areas located in the circum-oral region. This squamation pattern is found in schooling or shoaling species of sharks that swim at low to moderate speed (Fig 5C). In thelodonts, CVAs and/or qualitative interpretation of the data allow reliably identification of this distinctive squamation pattern in several species with articulated specimens (*Cometicercus talimaae*, *Drepanolepis maerssae*, *Lanarkia spinulosa*, *Nikolivia milesi*, *Pezopallichthys ritchiei*, *Shielia gibba*, *Shielia parca*, *Shielia taiti* and *Sphenonectris turnerae*; Fig 6C, 6D, 6L, 6R, 6S, 6X, 6Y, 6Z, 6A’ and 6B’).
3 Squamation patterns where the largest part of the body is covered with scales of generalized functions (morphotype 8). Abrasion resistant scales are present in a smaller proportion and restricted to the anterior-most part of the animal, usually on the snout and around the mouth. This squamation pattern is characteristic of slow species of sharks of the open water which are commonly associated to the outer continental shelf and continental slope (Fig 5G). In thelodonts, CVAs allow reliable recognition of this squamation pattern in complete specimens preserved in ventral or lateral view (*Lanarkia horrida*, *Loganellia scotica* and *Loganellia sulcata;* Fig 6J, 6O and 6Q, Table 7). However, the assignment of the species with specimens preserved in dorsal or in an indeterminate view (*Lanarkia lanceolata* and *Loganellia prolata;* Fig 6K–6M) was not conclusively as CVA-2.2 (considering only the squamation of the dorsal area of the body) does not properly differentiate between slow species of the open water or demersal species on soft substrates (Table 7). In any case, their possible assignments are restricted to one these two ecological groups. Special consideration deserves the cases of *Loganellia scotica* and *Lanarkia horrida*. The species *Loganellia scotica* is consistently assigned to slow species of the open water based on the specimen preserving the ventral squamation (CVA-2.3 in Table 7), but when the percentages of coverage of both ventral and dorsal areas are considered together, it is assigned to demersal species on sandy and muddy substrates (CVA-2.1 in Table 7). Nonetheless, the ventral squamation pattern of *Loganellia scotica* is in fact quite different to that present in the ventral body side of demersal species on soft substrate where abrasion resistant scales cover up to 100% whereas scales with generalized functions are completely absent in most cases or represent less than the 10% (41.9% and 31.4% in two isolated cases) (S8 Table). Contrarily, the ventral part of the body of *Loganellia scotica* is mostly covered with scales of generalized functions (61.9%) and scales for protection against abrasion (38.1%) are located only in the anterior-most part of the body (similar pattern is present in the dorsal part; Fig 6N and 6O and see also S13 Table), which is more consistent with its assignment to slow species of the open water (compare Fig 5E and 5G). Even so, the percentage of abrasion resistant scales covering the anterior body part of *Loganellia scotica* is significantly higher to that present in slow sharks of the open water (around 5%; S8 Table) as well as to the other articulated thelodonts assigned to the same ecological group (up to 15%; S14 Table). This peculiarity is indicative of a higher abrasion stress on the head and anterior trunk region that suggests the existence of some particular ecological aspects probably related with the feeding strategy. In this sense, digging behavior for detritivory could be a suitable explanation for this particular squamation pattern and probably for those of most thelodonts of the same ecological group that also show higher percentages of abrasion resistant scales than sharks. Interestingly, although some thelodonts have already been presupposed as detritivores [54], this feeding strategy could be specially widespread in this group typically associated to deep sea habitats (outer continental shelf and continental slope), where benthic detritus are a key component constituting the base of the trophic structure for the macrofauna [91]. On the other hand, the squamation pattern of *Lanarkia horrida* shows some features that make it unique among thelodonts. Large scales are interspersed together with smaller ones both on the dorsal and ventral part of the body (see [47]: Fig 34). Although trunk scales of all different sizes have been classified as scales of generalized function (morphotype 8) by the CVA-1 (S12 Table), Turner [11] suggested that enlarged scales of this species could be involved in defensive functions against predators, possibly being mobile or erectile. However comparable denticles present in living sharks and batoids seems to play a minor role in protection against predation [20]. As an interesting functional alternative, it has been revealed that elongate denticles of similar morphology are used in *Scyliorhinus canicula* for prey processing during the first stages of life [66]. Juveniles of this species rub large food items against the scales of the lateral-caudal region, obtaining smaller fragments that can be ingested (scale-rasp behavior). It is possible that *Lanarkia horrida* -and other thelodont species- used large elongated scales in similar prey processing behaviors, involving scale rasping (as suggested by Southall and Sims [66]) and offering an alternative to the microphagous feeding mechanism supposed by the lack of jaws.

Only in one articulated thelodont, *Thelodus laevis*, the squamation pattern does not show a clear correlation with any of those observed in extant sharks. The squamation of this species consists on a combination of abrasion resistant scales (with smooth crown surface) and scales of generalized functions (with strongly ornamented crowns) interspersed together on the trunk body area (Fig 6C’). Although scale of both functional types appear also together in the trunk area of demersal sharks species inhabiting on sandy and muddy substrates, their topological distribution is clearly different. Hence, whereas scales of both functional types occur well separated and restricted to specific body areas in extant sharks (Fig 5E), they are mixed together forming a mosaic pattern that makes it difficult to establish the boundaries between their distributions in *Thelodus laevis* (Fig 6C’). Märss [60] suggested that this characteristic combination of morphologies respond to an ontogenetic process where new ornamented scales are added between the smooth scales typical from early ontogenetic stages. Accordingly, the squamation of this specimen is probably an intermediate young stage between the juvenile squamation, completely covered with smooth scales, and adult squamation, completely covered with strongly ornamented scales. Ontogenetic changes have also been well documented in the squamations of *Lanarkia horrida* and *Loganellia scotica* [28, 47, 60].

In summary, the analysis of squamation patterns in articulated specimens of thelodonts provides important information regarding to the exact body distribution of scale morphotypes (and functional types) and to the quantification of the areas they occupy, allowing reliable assignments to one concrete ecological group for most of the species. Moreover, some other common features of the squamation of sharks can be recognized in articulated thelodonts. For example, closely packed rhomboidal scales on the leading edge of the fins are present in *Lanarkia horrida* (MB.f.3979) and some other species (see [3] and references therein); smooth rounded scales on the rostral area have been found in *Loganellia scotica* (MB.f.4012; see also [48]); and specialized scales around the branchial openings have also been recognized in thelodonts (see [47, 50, 51, 52, 53, 54, 57]).

However, as noted above, the vast majority of thelodont species (80%) are known exclusively on the basis of associations of disarticulated scales. For this reason, their study became necessary in order to obtain a complete view of the ecological diversity in the group. For the study of these species the body coverage percentages occupied by each topological series has been estimated taking two thelodont species (*Loganellia scotica* and *Furcacauda fredholmae*) as models. Obviously, although interspecific variation in these percentages would be expected to exist, the study of articulated specimens shows a significant interspecific homogeneity. No major variations were observed in any of the different species of non-furcacaudiform thelodonts consulted at the National Museum of Scotland (Edinburgh, Scotland) and the Museum für Naturkunde (Berlin, Germany) and those studied from the literature. Similarly, a clear uniformity also exists in all furcacaudiform species known from articulated remains (See [54]). Anyway, slight variations of these percentages would have minimum effect on classification results as it is evidenced by the variation found in the coverage percentage of each scale morphotype in sharks of the same ecological group (S8 Table).

When thelodont species know from disarticulated remains are included in the CVA-2 or analyzed qualitatively the largest part of them are assigned to the three ecological groups identified in articulated specimens (S16 Table and see also S1 Appendix), however two new squamation patterns are recognized:

4 Squamation patterns where the largest part of the body is covered with drag reduction scales (morphotype 5). In sharks, this morphotype is exclusive of strong-swimming species (i.e., large near-shore hunters and fast pelagic hunting species, according Reif [15]; Fig 5A and 5B). Scales of this morphotyope have been found in ten species of thelodonts (*Praetrilogania grabion*, *Trimerolepis gemella*, *Thelodus visvaldi*, *Apalolepis obruchevi*, *A*. *angelica*, *A*. *brotzeni*, *Skamolepis fragilis*, *Canonia grossi*, *C*. *costulata* and *C*. *kaerberi*; S15 Table), all of them being reliably assigned to this ecological group on the basis of CVA-1 or qualitative interpretation (S16 Table and see also S1 Appendix). In all the cases the average of the distance between the ridges of the crown surface is within 35–80 μm approximately and this value is almost constant for the scales of each species. This evidences a clear involvement in drag reduction and demonstrates a clear morphological convergence with scales of fast pelagic hunting sharks (compare in Table 8; see also [15, 16, 19, 20]). Our results further suggest that the same strategies that allow extant fast pelagic hunting sharks to keep constant the distance between ridges (see above) had already evolved during the Paleozoic in thelodonts. Some pelagic thelodonts kept this distance within narrow limits maintaining constant both the number of ridges and the crown width. In thelodonts that follow this strategy, crown width has low standard deviation values (e.g. *Canonia kaerberi* in Table 8; CW SD = 25.2 μm, CW CV = 11.0%). On the contrary, other pelagic thelodont species show a strong variability in crown width but maintain constant distances between ridges by adding further ridges in wider scales. In species that follow this second strategy, crown width has high standard deviation values (e.g. *Thelodus visvaldi* in Table 8; CW SD = 185.1 μm, CW CV = 44.0%) and no significant correlation is found between the average of distance between ridges and the crown width (Sig. 0.602). Interestingly, scale crown width of two apalolepids (*Apalolepis obruchevi* and *Apalolepis angelica*) clearly exceeds the typical rage of values observed for pelagic shark scales (Table 8). Even so, drag reduction function is also expected for these unusual big scales as their average ridge distances are comprised within the optimal functional values varying between a narrow range (ARD mean = 83.5 μm and ARD SD = 7.5 μm for *Apalolepis obruchevi;* ARD mean = 38.0 μm and ARD SD = 8.0 μm for *Apalolepis angelica*; compare with [15, 16, 19, 20]). Moreover, the presence of scales with thinner crowns is another common pattern documented in extant pelagic sharks for encouraging a greater hydrodynamic efficiency [20]. Similarly a trend in scale crown thinning was documented in apalolepids [3], a group classified here mostly within a pelagic lifestyle. Altogether, evidences point towards the existence of highly specialized drag-reduction scales in thelodonts, analogous to those present in fast pelagic hunting sharks. Although, obviously, the lack of jaws in thelodonts precludes a macropredatory hunting habit, the presence of this functional clearly suggest that some species were well adapted to a rapid strong swimming.
5 Squamation patterns characterized by the co-occurrence of abrasion resistant scales (morphotypes 6 or 7) and scales with generalized functions (morphotype 8) in the trunk region. This combination is characteristic of demersal sharks on sandy and muddy substrates where abrasion resistant scales are restricted to the ventral region and scales with generalized functions are mainly confined to the dorsal side (Fig 5E). Nine thelodont species known from isolated scales (*Loganellia cuneata*, *L*. *exilis*, *L*. *unispinata*, *Overia adraini*, *Thelodus carinatus*, *T*. *marginatus*, *T*. *matukhini*, *Turinia composita*, *T*. *gondwana*; S15 Table) show this same combination but the exact topological distribution of both scale functional types cannot be determined. In this sense, abrasion resistant scales and scales with generalized functions have also been reported in combination in one articulated thelodont (*Thelodus laevis*), however their topological distribution differs considerably from that observed in extant sharks being probably a transitional steep of the squamation of juveniles and adults (see above). Therefore, similar ontogenetic changes cannot be discarded for other thelodont species and scale assemblages with both functional types could be indeed a mixture of scales from different ontogenetic stages. However, these species have been tentatively considered as demersal thelodonts on soft substrates here (S1 Appendix), waiting for further studies and the discovery of more articulated specimens that help to clarify this question.
  As indicated above, scales of *Longodus acicularis* were classified by CVA-1 into scales that enable bioluminescence (morphotypes 2 and 3; S15 Table). Scales of *Longodus acicularis* are bristle shaped and shows very high length/width ratio, extremely similar in fact to the values measured on scales of *Etmopterus spinax* and other bioluminescent species (Compare S5 Table and S15 Table). However, scales of bioluminescent groups exhibit some other morphological features that make them easily distinguishable [15, 61]. In this sense, the crown angle in *Longodus acicularis* scales differs considerably from that of the typical scales of bioluminescent sharks, thus not being suitable for the passage of the light emitted by the photophores. Furthermore, sedimentological data suggest that *Longodus acicularis* remains come from shallow lagoonal environments [92], in no case, compatible with the existence of an aphotic zone. As a consequence, it seems more prudent to keep the ecological assignment of *Longodus acicularis* as uncertain waiting for the discovery of more disarticulated or articulated remains.

**Fig 8:**
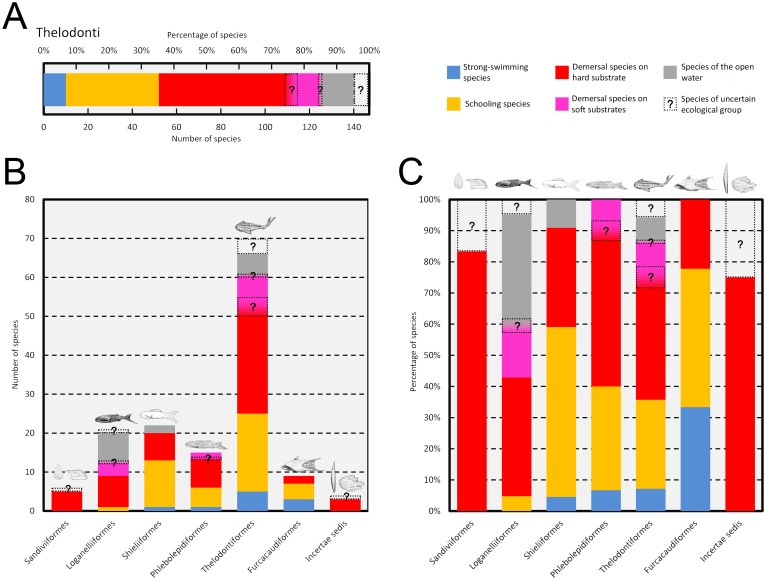
Summary of the ecological diversity present in thelodonts including both CVA and qualitative assignments. Bar charts show the absolute number and percentage of species assigned to each ecological group considering all species of thelodonts both together (A) and arranged by the currently described orders (B and C). Dashed rectangle outlines with question mark represent a number or a percentage of species whose assignment is uncertain. Uncertainties situated between two ecological groups correspond to species that belong to one of these two groups but a definitive assignment is not possible without further data. Uncertainties situated at the top of the bar represent species that could belong to any of the ecological groups. Drawings of *Larolepis darbyi* scales (Sandiviiformes?), *Loganellia scotica* (Loganelliiformes), *Shielia taiti* (Shieliiformes), *Phlebolepis elegans* (Phlebolepidiformes), *Lanarkia horrida* (Thelodontiformes), *Furcacauda fredholmae* (Furcacaudiformes), *Longodus acicularis* scales (*Incertae sedis*) and scales of *Thulolepis striaspina* (*Incertae sedis*) taken from Sansom and Elliott [93], Halstead and Turner [94], Märss and Ritchie [47], Ritchie [95], Turner [11], Wilson and Caldwell [58], Märss [92] and Blom [78] respectively.

In sum, te study of the squamation of thelodonts reveals the presence of a remarkable diversity of lifestyles and habitat preferences in the group (Fig 8), including at least four well recognized ecological groups: (1) demersal thelodonts that lived on hard substrates (57 species, 39% of the total), (2) shoaling or schooling thelodonts of low to moderate speed (42 species, 29% of the total), (3) slow thelodont that inhabited the open water (14 species, 10% of the total), and (4) pelagic swimming specialist thelodonts (10 species, 7% of the total). Additionally, a fifth different squamation pattern is identified and tentatively assigned to (5) demersal species on sandy and muddy substrates (9 species, 6% of the total). Note that the ecological assignment of a few number of species is not conclusive remaining with uncertainties. Six species (4% of the total) could pertain to demersal species on sandy and muddy substrates or to demersal species on hard substrates; two species (1% of the total) could pertain to slow species of the open water or demersal species on sandy and muddy substrates; and the ecological group of seven species (5% of the total) remains unknown.

Thelodonts with squamations consistent with demersal lifestyles associated to hard bottoms, probably in abrupt rocky nearshore environments with grooves and small caves, or associated to reefs, are the most abundant, being well represented in all major lineages of thelodonts from the Ordovician to the Devonian. This together with the fact that all Sandiviiformes (the oldest putative thelodont lineage) belong to this ecological group suggest that protection against abrasion probably was the plesiomorphic function of the squamation in thelodonts. These habitats provides protection against predators, but this requires of a relatively flexible bodies that allow seeking refuge within caves and crevices or even among the different organisms that form the reef structure. Interestingly, most of the other contemporary agnathan groups present well developed rigid cephalic shields, usually, with non-articulated protruding plates (i.e., dorsal, cornual, etc). The micromeric nature of the exosqueleton in thelodonts could allow them to be flexible enough for inhabiting more closed environments such as rocky nearshore and reefs whereas the flexibility and maneuverability of other ‘ostracoderms’ was limited, being the latter ones probably restricted to other more open habitats. This contrasts with some previous reconstructions placing thelodonts on sandy or muddy substrates (e.g. [54, 96]). In fact, our analyses show that only few species of thelodonts can be tentatively included into demersal species on sandy and muddy substrates (nine species, constituting 6% of the total). Schooling or shoaling species of low to moderate speed were probably well represented in phlebolepidiforms and shieliiforms during the Silurian and in thelodontiforms and furcacaudiforms during the Devonian. This finding has some interesting implications. Advantages for migration, predation risk, forage selection, cooperative hunting, activity budget, thermal niche–fecundity and social factors have been proposed as possible drives for the evolution of grouping behaviour in extant sharks (see Jacoby et al. [97] and references therein). In consequence, it stands to reason that at least some of these factors promoted social interactions also among thelodonts during the Paleozoic. On the other hand, some costs associated with grouping behaviour such as the increase of the risk of parasite transmission have also been described in extant fishes (e.g. Richards et al. [98]). Body surface of schooling or shoaling sharks is mainly covered by scales with defensive functions against ecotoparasites. The presence of this particular functional type in phylogenetically and temporally distant thelodont species suggests that ecotoparasitism could be an important pressure selection since very early stages in the evolution of vertebrates. In fact, hook circlets have been described in acanthodians and placoderms from the Devonian of Latvia constituting a potential fossil evidence of parasitic flatworms in basal gnathostomes (see De Baets et al. [99] and references therein). Thelodonts with squamations that suggest slow swimming in the open water are well represented in loganelliiforms (nearly 35% of them). Supporting this assignment, several species of this order have been found in presumed turbiditic sediments suggesting a close relationship with the outer continental shelf or the slope [3]. Probably, one of the most interesting findings of this work is the identification of several thelodonts with squamations typical of pelagic swimming specialists, well represented among thelodontiforms and furcacaudiforms. Strikingly, the adaptation to a pelagic lifestyle could have evolved almost simultaneously in different thelodont lineages during the Early Devonian, when planktonic food availability and competition in the diversity-saturated benthic habitats enabled the colonization of the water column by many other groups, the so called Devonian Nekton Revolution [100].

## Supporting information

S1 Table Summary details for all studied shark specimens. Taxonomic identification, registration number, sex determination and total length are given for each specimen. Length at birth and maturity are also provided for each species.
(XLSX)

S2 Table Summary details for all articulated thelodonts studied first hand. Taxonomic identification and registration number are given for each specimen.
(XLSX)

S3 Table Summary details for articulated thelodonts. Taxonomic identification, registration number and details of the degree and position of preservation are given for each specimen.
(XLSX)

S4 Table Summary details for all studied thelodont scales coming from disarticulated remains. Taxonomic identification and topological series are given for each scale.
(XLSX)

S5 Table Numerical values for all variables measured on shark scales of known morphotype and functional type. (XLSX)

S6 Table Results of Canonical Variate Analysis-1 (CVA-1, with eight scale morphotypes as defined groups and ten size-free variables of the crown surface as discriminant variables). (A) Wilks' lambda test of functions. (B) Pairwise group comparisons. (C) Detailed results of the original and cross-validated classification for all scales of known morphotype and functional type. The assignment probabilities for the highest and the second highest predicted morphotype are given for each scale.
(XLSX)

S7 Table Numerical values for discriminant variables measured on shark scales of unknown morphotype (and functional type) and detailed results of the classification after their inclusion in Canonical Variate Analysis-1 (CVA-1, with eight scale morphotypes as defined groups and ten size-free variables of the crown surface as discriminant variables). (XLSX)

S8 Table Percentage of coverage of each scale morphotype and functional type on the whole body, dorsal side and ventral side of the body of the studied sharks arranged by ecological groups. Fast pelagic hunting species and large near-shore hunters were reunified into strong-swimming pelagic species for CVA-2. It has been considered as dorsal side of the body the dorso-lateral region, the dorsal fins, the dorsal surface of pectoral and pelvic fins and the caudal fin; and as ventral side of the body the ventral region, the ventral surface of the pectoral and pelvic fins and the caudal fin. The caudal fin surface has been considered in both cases because it is usually preserved in lateral view in thelodonts.
(XLSX)

S9 Table Results of Canonical Variate Analysis-2 (CVA-2, with six ecological groups as defined groups and scale morphotype percentages of coverage on the whole body as discriminant variables). (A) Wilks' lambda test of functions. (B) Pairwise group comparisons. (C) Detailed results of the original and cross-validated classification for all sharks. The assignment probabilities for the highest and the second highest predicted ecological group are given for each specimen.
(XLSX)

S10 Table Results of Canonical Variate Analysis-2.2 (CVA-2.2, with six ecological groups as defined groups and scale morphotype percentages of coverage on the dorsal side of the body as discriminant variables). (A) Summary statistics. Eigenvalues, proportions of explained variance, canonical correlations and standardized coefficients are shown for each discriminant function. (B) Wilks' lambda test of functions. (C) Pairwise group comparisons. (D) Summary results of the original and cross-validated classification for all studied sharks. (E) Detailed results of the original and cross-validated classification for all sharks. The assignment probabilities for the highest and the second highest predicted ecological group are given for each specimen.
(XLSX)

S11 Table Results of Canonical Variate Analysis-2.3 (CVA-2.3, with six ecological groups as defined groups and scale morphotype percentages of coverage on the ventral side of the body as discriminant variables). (A) Summary statistics. Eigenvalues, proportions of explained variance, canonical correlations and standardized coefficients are shown for each discriminant function. (B) Wilks' lambda test of functions. (C) Pairwise group comparisons. (D) Summary results of the original and cross-validated classification for all studied sharks. (E) Detailed results of the original and cross-validated classification for all sharks. The assignment probabilities for the highest and the second highest predicted ecological group are given for each specimen.
(XLSX)

S12 Table Numerical values for discriminant variables measured on thelodont scales coming from articulated remains and detailed results of the classification after their inclusion in Canonical Variate Analysis-1 (CVA-1, with eight scale morphotypes as defined groups and ten size-free variables of the crown surface as discriminant variables). (XLSX)

S13 Table Percentage of coverage of each scale morphotype and functional type in all articulated thelodonts. (XLSX)

S14 Table Detailed results of the classification for all articulated thelodonts after their inclusion in Canonical Variate Analyzes-2.1–3 (CVA-2.1–3, with six ecological groups as defined groups and scale morphotype percentages of coverage on the whole body, dorsal side or ventral side of the body as discriminant variables respectively). (XLSX)

S15 Table Numerical values for discriminant variables measured on thelodont scales coming from disarticulated remains and detailed results of the classification after their inclusion in Canonical Variate Analysis-1 (CVA-1, with eight scale morphotypes as defined groups and ten size-free variables of the crown surface as discriminant variables). (XLSX)

S16 Table Estimated percentage of coverage of each morphotype and functional type in all thelodonts known from disarticulated remains and detailed results of the classification after their inclusion in Canonical Variate Analysis-2 (CVA-2, with six ecological groups as defined groups and scale morphotype percentages of coverage on the whole body as discriminant variables). (XLSX)

S1 Text Supporting information references. (DOCX)

S1 Appendix Ecological diversity in thelodonts. Compilation of all described species of thelodonts and the assigned ecological group with indication of body coverage percentages of each scale morphotype and functional type, details of the specimens preservation (CS, complete squamation; DR, desarticulated remains; PS, partial squamation; SP, scale patch; d, dorsal view; dv, dorsal and ventral view; v, ventral view; i, indeterminate view; l, lateral view) and type of assignment process (CVA, qualitative interpretation or both). Remains that allowed the ecological group assignment of each species are indicated in boldface.
(DOCX)

## References

[1] Sansom IJ, Smith MM, Smith MP. Scales of thelodont and shark-like fishes from the Ordovician of Colorado. Nature. 1996;379: 628–629.

[2] Märss T., Karatajûte−Talimaa V. Ordovician and Lower Silurian thelodonts from Severnaya Zemlya Archipelago (Russia). Geodiversitas. 2002;24: 381–404.

[3] Märss T, Turner S, Karatajûte−Talimaa V. Handbook of Palaeoichthyology, Volume 1B, Agnatha II. Verlag Dr. Friedrich Pfeil, Munchen; 2007.

[4] Turner S, Hairapetian V. Thelodonts from Gondwana. Ichthyolith Issues Special Publication. 2005;8: 24–28.

[5] Hairapetian V, Roelofs BP, Trinajstic KM, Turner S. Famennian survivor turiniid thelodonts of North and East Gondwana. Geological Society, London, Special Publications. 2015;423: SP423. 3.

[6] Turner S. Siluro-Devonian thelodonts from the Welsh Borderland. Journal of the Geological Society. 1973;129: 557–582.

[7] Turner S. Sequence of Devonian thelodont scale assemblages in East Gondwana. Geological Society of America Special Papers. 1997;321: 1–45.

[8] Märss T, Fredholm D, Karatajûte−Talimaa V, Turner S, Jeppsson L, Nowlan G. Silurian vertebrate biozonal scheme. Geobios. 1995;19: 368–372.

[9] Märss T, Fredholm D, Karatajûte−Talimaa V, Turner S, Jeppsson L, and Nowlan G. Towards the Silurian vertebrate biozonal standard. In: Johnson ME, Brett CE, editors. The James Hall Symposium: Second International Symposium on the Silurian System, Program and Abstracts. University of Rochester, New York; 1996. pp. 73–74.

[10] Blieck A, Turner S, Young GC, Luksevics E, Mark-Kurik E, Talimaa VN, et al Devonian vertebrate biochronology and global marine/non-marine correlation. Courier-Forschungsinstitut Senckenberg. 2000; 161–194.

[11] Turner S. Thelodont lifestyles In: Mark-Kurik E, editor. Fossil Fishes as Living Animals; 1992 pp. 21–40.

[12] Turner S. Early Silurian to Early Devonian thelodont assemblages and their possible ecological significance In: Boucot AJ, Lawson J, editors. Palaeocommunities: A Case Study From the Silurian and Lower Devonian; 1999 pp. 42–78.

[13] Muñoz-Chápuli R. Sobre la clasificación tipológica del esqueleto dérmico de escualos. Miscel·lànea Zoològica. 1985;9: 396–400.

[14] Reif WE. Morphogenesis and function of the squamation in sharks. Comparative functional morphology of shark scales, and ecology of sharks. Neues Jahrbuch für Geologie und Paläontologie, Abhandlungen. 1982;164: 172–183.

[15] Reif WE. Squamation and ecology of sharks. Courier Forschungsinstitut Senckenberg. 1985;78: 1–255.

[16] Reif WE., Dinkelacker A. Hydrodynamics of the squamation in fast swimming sharks. Neues Jahrbuch für Geologie und Paläontologie, Abhandlungen. 1982;164: 184–187.

[17] Fulgosi FC, Gandolfi G. Re-description of the external morphology of Somniosus rostratus (Risso, 1826), with special reference to its squamation and cutaneous sensory organs, and aspects of their functional morphology (Pisces Selachii Squalidae). Monitore Zoologico Italiano-Italian Journal of Zoology. 1983;17: 27–70.

[18] Bechert DW, Hoppe G, Reif W-E. On the drag reduction of the shark skin. 1985 American Institute of Aeronautics and Astronautics Shear Flow Control Conference. 1985.

[19] Raschi W, Musick J. Hydrodynamic aspects of shark scales. NASA Contractor Report. 1986; 3963.

[20] Raschi W, Tabit C. Functional aspects of placoid scales: a review and update. Australian Journal of Marine & Freshwater Research. 1992;43: 123–147.

[21] Reif WE. Protective and hydrodynamic function of the dermal skeleton of elasmobranchs. Neues Jahrbuch für Geologie und Paläontologie, Abhandlungen. 1978;157: 133–141.

[22] Ball P. Engineering shark skin and other solutions. Nature. 1999;400: 507–509.

[23] Turner S. Thelodonts and the Silurian-Devonian boundary. Journal of the Geological Society. London. 1971;127: 632–35.

[24] Turner S. The nature of dentine scales. A study of dermal denticles—comparison of thelodont and shark scales. VPCA University of Newcastle upon Tyne. 1977.

[25] Turner S. A new articulated thelodont (Agnatha) from the Early Devonian of Britain. Palaeontology. 1982;25: 879–89.

[26] Reif WE. Types of morphogenesis of the dermal skeleton in fossil sharks. Paläontologische Zeitschrift. 1978;52: 110–128.

[27] Karatajûte−Talimaa V. Determination methods for the exoskeletal remains of early vertebrates. Fossil Record. 1998;1: 21–51.

[28] Turner S. Monophyly and interrelationships of the Thelodonti In: Chang MM, Liu YH, Zhang GR, editors. Early vertebrates and related problems of evolutionary biology; 1991 pp. 87–119.

[29] Janvier P. Early vertebrates. Oxford: Clarendon Press; 1996.

[30] Traquair RH. On *Thelodus pagei* Powrie, sp. from the Old Red Sandstone of Forfarshire. Transactions of the Royal Society of Edinburgh. 1899;39: 595–602.

[31] Traquair RH. Report on fossil fishes collected by the Geological Survey of Scotland in the Silurian rocks of the south of Scotland. Transactions of the Royal Society of Edinburgh. 1899;39: 827–864.

[32] Traquair RH. The bearings of fossil ichthyology on the problem of evolution. Geological Magazine. 1900;7: 463–470.

[33] Traquair RH. Supplementary report on fossil fishes collected by the Geological Survey of Scotland in Upper Silurian rocks of Scotland. Transactions of the Royal Society of Edinburgh. 1905;40: 879–888.

[34] Woodward AS. Catalogue of Fossil Fishes in the British Museum (Natural History) Part II. Elasmobranchi (Acanthodii), Holocephali, Ichthyodurolites, Ostracodermi, Dipnoi and Teleostomi (Crossopterygii and Chondrostean Actinopterygii). British Museum (Natural History), London; 1891.

[35] Rohon JV. Die obersilurische Fische von Oesel. I. Theil: Thyestidae und Tremataspidae. Mémoires de l'Académie Impériale des Sciences de St. Pétersbourg. 1892;38: 1–88.

[36] Rohon JV. Über untersilurische Fische. Bulletin de l’Académie Impériale des Sciences de St.-Pétersbourg, Nouvelle Série. 1892; 269–277.

[37] Rohon JV. Die obersilurischen Fische von Oesel. II. Theil: Selachii, Dipnoi, Ganoidei, Pteraspidae and Cephalaspidae. Mémoires de l'Académie Impériale des Sciences de St. Pétersbourg. 1893;41: 1–124.

[38] Märss T. Squamation of the thelodont agnathan *Phlebolepis*. Journal of Vertebrate Paleontology. 1986;6: 1–11.

[39] Hairapetian V, Blom H, Miller CG. Silurian thelodonts from the Niur Formation, central Iran. Acta Palaeontologica Polonica. 2008;53: 85–95.

[40] Žigaitė Z. Endemic thelodonts (Vertebrata: Thelodonti) from the Lower Silurian of central Asia and southern Siberia. Earth and Environmental Science Transactions of the Royal Society of Edinburgh. 2013;104: 1–21.

[41] Žigaitė Z, Karatajûte−Talimaa V, Goujet D, Blom H. Thelodont scales from the Lower and Middle Devonian Andrée Land Group, Spitsbergen. 2013;135: 57–73.

[42] Gross W. Über Thelodontier-Schuppen. Palaeontographica Abteilung A. 1967; 1–67.

[43] Karatajûte−Talimaa V. Silurian and Devonian thelodonts of the USSR and Spitsbergen Translation Bureau; 1978.

[44] Turner S. Thelodonti (Agnatha) In: Westphal F, editor. Fossilium Catalogus, 1: Animalia; 1976 pp. 1–35.

[45] Botella H, Valenzuela-Ríos JI, Carls P. A new Early Devonian thelodont from Celtiberia (Spain), with a revision of Spanish thelodonts. Palaeontology. 2006;49: 141–154.

[46] Märss T. *Thelodus admirabilis* n. sp. (Agnatha) from the Upper Silurian of the East Baltic. Eesti NSV Teaduste Akadeemia Toimetised Geoloogia. 1982;31: 112–116.

[47] Märss T., Ritchie A. Articulated thelodonts (Agnatha) of Scotland. Transactions of the Royal Society of Edinburgh, Earth Sciences. 1998;88: 143–195.

[48] Žigaitė Z, Goujet D. New observations on the squamation patterns of articulated specimens of *Loganellia scotica* (Traquair, 1898) (Vertebrata: Thelodonti) from the Lower Silurian of Scotland. Geodiversitas. 2012;34: 253–270.

[49] Wilson MVH, Märss T. Anatomy of the Silurian thelodont *Phlebolepis elegans* Pander. Estonian Journal of Earth Sciences. 2012;61: 261–276.

[50] Van der Brugghen W, Janvier P. Denticles in thelodonts. Nature. 1993;364: 107.7686630

[51] Turner S. Remarks on the early history of chondrichthyans, thelodonts, and some “higher elasmobranchs”. New Zealand. Geological Survey Record. 1985;9: 93–95.

[52] Turner S. Thelodont squamation. Ichthyolith Issues. 1994;13: 12–15.

[53] Turner S. New Llandovery to early Pridoli microvertebrates including Lower Silurian zone fossil, Loganellia avonia nov. sp., from Britain. Courier-Forschungsinstitut Senckenberg. 2000; 91–128.

[54] Wilson MVH, Caldwell MW. The Furcacaudiformes: A new order of jawless vertebrates with thelodont scales, based on articulated Silurian and Devonian fossils from northern Canada. Journal of Vertebrate Paleontology. 1998;18: 10–29.

[55] Hout W in't. Thelodonten, een raadselachtige groep vissen Grondboor en Hamer. 1990; 14–10.

[56] Hout W in't. Thelodonts, an enigmatic group of fish Ichthyolith Issues. 1990b;5: 21–30.

[57] Märss T, Wilson MVH, Thorsteinsson R. Silurian and Lower Devonian thelodonts and putative chondrichthyans from the Canadian Arctic Archipelago (Cornwallis, Baillie-Hamilton, Devon, and Prince of Wales islands). Special Papers in Palaeontology. 2006;75: 1–140.

[58] Wilson MVH, Caldwell MW. New Silurian and Devonian fork-tailed ‘thelodonts’ are jawless vertebrates with stomachs and deep bodies. Nature. 1993;361: 442–444.

[59] Caldwell MW, Wilson MV. Comparison of the body formand squamation of “fork-tailed” agnathans with that of conventional thelodonts. Geobios. 1995;28: 23–29.

[60] Märss T. A unique Late Silurian *Thelodus* squamation from Saaremaa (Estonia) and its ontogenetic development. Estonian Journal of Earth Sciences. 2011;60: 137–146.

[61] Reif WE. Functions of scales and photophores in mesopelagic luminescent sharks. Acta Zoologica (Stockholm). 1985;66: 111–118.

[62] Compagno LJV. Sharks of the world. An annotated and illustrated catalogue of sharks species known to date. Hexanchiformes to Lamniformes. FAO Fisheries Synopsis, Volume 4, Part 1; 1984.

[63] Compagno LJV. Sharks of the world. An annotated and illustrated catalogue of shark species known to date. Carcharhiniformes. FAO Fisheries Synopsis, Volume 4, Part 2; 1984.

[64] Compagno LJV. Sharks of the world. An annotated and illustrated catalogue of shark species known to date. Bullhead, mackerel and carpet sharks. Heterodontiformes, Lamniformes and Orectolobiformes. FAO Fisheries Synopsis, Volume 2; 2001.

[65] Grover CA. Juvenile denticles of the swell shark Cephaloscyllium ventriosum: function in hatching. Canadian Journal of Zoology. 1974;52: 359–363.

[66] Southall EJ, Sims DW. Shark skin: a function in feeding. Proceedings of the Royal Society of London, Series B (Suppl.). 2003;270: 47–49. 10.1098/rsbl.2003.0006 PMC1698022 12952633

[67] Mojetta A. Guía del mundo submarino: Tiburones. Editorial Diana; 2005.

[68] Atkinson CJ, Collin SP. Structure and topographic distribution of oral denticles in elasmobranch fishes. The Biological Bulletin. 2012;222: 26–34. 10.1086/BBLv222n1p26 22426629

[69] Ferrón H, Pla C, Martínez-Pérez C, Escudero-Mozo MJ, Botella H. Morphometric Discriminant Analysis of isolated chondrichthyan scales for palaeoecological inferences: the Middle Triassic of the Iberian Chain (Spain) as a case of study. Journal of Iberian Geology. 2014;40: 87–97.

[70] Ciena AP, S Rangel B, Bruno CEM, Miglino MA, Amorim AF, Rici REG, et al Morphological aspects of oral denticles in the Sharpnose shark Rhizoprionodon lalandii (Müller and Henle, 1839)(Elasmobranchii, Carcharhinidae). Anatomia, histologia, embryologia. 2015;45: 109–114. 10.1111/ahe.12178 25898917

[71] Albrecht GH. Multivariate analysis and the study of form, with special reference to canonical variate analysis. American Zoologist. 1980;20: 679–693.

[72] Shark Trust. An Illustrated Compendium of Sharks, Skates, Rays and Chimaera. Chapter 1: The British Isles and Northeast Atlantic. Part 2: Sharks. 2010

[73] Compagno LJV, Dando M, Fowler S. Sharks of the world, 368 pp. Princeton University Press, New Jersey; 2005.

[74] Froese R, Pauly D. FishBase. World Wide Web electronic publication. Version (04/2013). 2013. www.fishbase.org.

[75] Ebert D, Fowler S, Compagno L. Sharks of the world. A fully illustrated guide. Wild Nature Press, Plymouth; 2013.

[76] IUCN. The IUCN Red List of Threatened Species. Version 2015–4. 2015. http://www.iucnredlist.org.

[77] Olaso I, Velasco F, Serrano A, Rodríguez-Cabello C, Cendrero O. Trophic Relations of Lesser-Spotted catshark (*Scyliorhinus canicula*) and Blackmouth Catshark (*Galeus melastomus*) in the Cantabrian Sea. Journal of Northwest Atlantic Fishery Science. 2004;35: 481–494.

[78] Blom H. *Loganellia* (Thelodonti, Agnatha) from the Lower Silurian of North Greenland. Acta Geologica Polonica. 1999;49: 97–104.

[79] Karatajûte-Talimaa V, Märss T. Upper Silurian thelodonts from Severnaya Zemlya Archipelago (Russia). Geodiversitas. 2002;24: 405–443.

[80] Blom H, Goujet D. Thelodont scales from the Lower Devonian Red Bay Group, Spitsbergen. Palaeontology. 2002;45: 795–820.

[81] Karatajûte-Talimaa V. Lower Devonian (Lochkovian) thelodonts from the October Revolution Island (Severnaya Zemlya Archipelago, Russia). Geodiversitas. 2002;24: 791–804.

[82] Compagno LJV. Alternative life-history styles of cartilaginous fishes in time and space. Environmental Biology of Fishes, 1990;28: 33–75.

[83] Harvey PH, Pagel MD. The comparative method in evolutionary biology. Oxford university press, Oxford; 1991.

[84] Ricklefs RE, Miles DB. Ecological and evolutionary inferences from morphology: an ecological perspective In: Wainwright PC, Reilly SM, editors. Ecological morphology: integrative organismal biology; 1994 pp. 13–41.

[85] Vélez-Zuazo X, Agnarsson I. Shark tales: a molecular species-level phylogeny of sharks (Selachimorpha, Chondrichthyes). Molecular Phylogenetics and Evolution. 2011;58: 207–217. 10.1016/j.ympev.2010.11.018 21129490

[86] Motta P, Habegger ML, Lang A, Hueter R, Davis J. Scale morphology and flexibility in the shortfin mako *Isurus oxyrinchus* and the blacktip shark *Carcharhinus limbatus*. Journal of morphology. 2012;273: 1096–1110. 10.1002/jmor.20047 22730019

[87] Dean B, Bhushan B. Shark-skin surfaces for fluid-drag reduction in turbulent flow: a review. Philosophical Transactions of the Royal Society of London A: Mathematical, Physical and Engineering Sciences. 2010;368: 4775–4806. 10.1098/rsta.2010.0201 20855320

[88] Lang AW, Hidalgo P, Motta PJ, Westcott M. Bristled shark skin: a microgeometry for boundary layer control?. Bioinspiration and Biomimetics. 2008;3: 046005 10.1088/1748-3182/3/4/046005 18838758

[89] García-Mayoral R, Jiménez J. Drag reduction by riblets. Philosophical Transactions of the Royal Society of London A: Mathematical, Physical and Engineering Sciences. 2011;369: 1412–1427. 10.1098/rsta.2010.0359 21382822

[90] Oeffner J, Lauder GV. The hydrodynamic function of shark skin and two biomimetic applications. Journal of Experimental Biology. 2012;215: 785–795. 10.1242/jeb.063040 22323201

[91] Rex MA. Community structure in the deep-sea benthos. Annual Review of Ecology and Systematics. 1981;12: 331–353.

[92] Märss T. Thelodonts (Agnatha) from the basal beds of the Kuressaare Stage, Ludlow, Upper Silurian of Estonia. Proceedings of the Estonian Academy of Sciences, Geology. 2006;55: 43–66.

[93] Sansom IJ, Elliott DK. A thelodont from the Ordovician of Canada. Journal of Vertebrate Paleontology. 2002;22: 867–870.

[94] Halstead LB, Turner S. Silurian and Devonian ostracoderms. Atlas of palaeobiogeography. 1973;53: 67–79.

[95] Ritchie A. Phlebolepis elegans, an Upper Silurian thelodont from Oesel, with remarks on the morphology of thelodonts. In: Ørvig T, editor. Current Problems in Lower Vertebrate Phylogeny, Nobel Symposium; 1968. pp. 81–88.

[96] Turner S. Studies on Palaeozoic Thelodonti (Craniata: Agnatha). Ph.D. thesis, University of Newcastle upon Tyne. 1984.

[97] Jacoby DM, Croft DP, Sims DW. Social behaviour in sharks and rays: analysis, patterns and implications for conservation. Fish and Fisheries. 2012;13: 399–417.

[98] Richards EL, van Oosterhout C, Cable J. Sex-specific differences in shoaling affect parasite transmission in guppies. PLoS ONE. 2010;5: e13285 10.1371/journal.pone.0013285 20949014 PMC2952601

[99] De Baets K, Dentzien-Dias P, Upeniece I, Verneau O, Donoghue PC. Chapter Three-Constraining the deep origin of parasitic flatworms and host-interactions with fossil evidence. advances in parasitology. 2015;90: 93–135. 26597066 10.1016/bs.apar.2015.06.002

[100] Klug C, Kroeger B, Kiessling W, Mullins GL, Servais T, Frýda J, et al The Devonian nekton revolution. Lethaia. 2010;43: 465–477.

