# Supplementary material for: Squamation and ecology of thelodonts
Source: PLoS One. 2017 Feb 27;12(2):e0172781. doi: 10.1371/journal.pone.0172781 (PMC5328365; doi:10.1371/journal.pone.0172781)
Supplement: S1 Text — (DOCX) [file pone.0172781.s017.docx]

**S1 Text. Supporting information references**

1. IUCN. The IUCN Red List of Threatened Species. Version 2015-4. 2015. Available : http://www.iucnredlist.org.

2. Froese R, Pauly D. FishBase. World Wide Web electronic publication. Version (04/2013). 2013. Available: www.fishbase.org.

3. Ebert D, Fowler S, Compagno L. Sharks of the world. A fully illustrated guide. Wild Nature Press, Plymouth; 2013.

4. Märss T, Ritchie A. Articulated thelodonts (Agnatha) of Scotland. Transactions of the Royal Society of Edinburgh, Earth Sciences. 1998;88: 143–195.

5. Märss T, Wilson MVH, Thorsteinsson R. Silurian and Lower Devonian thelodonts and putative chondrichthyans from the Canadian Arctic Archipelago (Cornwallis, Baillie-Hamilton, Devon, and Prince of Wales islands). Special Papers in Palaeontology. 2006;75: 1–140.

6. Märss T, Wilson MV, Thorsteinsson R. New thelodont (Agnatha) and possible chondrichthyan (Gnathostomata) taxa established in the Silurian and Lower Devonian of the Canadian Arctic Archipelago. Proceedings of the Estonian Academy of Sciences, Geology. 2002;51 :88–120.

7. Märss T. Squamation of the thelodont agnathan *Phlebolepis*. Journal of Vertebrate Paleontology. 1986;6: 1–11.

8. Märss T. A unique Late Silurian *Thelodus* squamation from Saaremaa (Estonia) and its ontogenetic development. Estonian Journal of Earth Sciences. 2011;60: 137–146.

9. Turner S. Vertebrate fauna of the Silverband Formation, Grampians, western Australia. Proceedings of the Royal Society of Victoria. 1986;98: 56–62.

10. Soehn KL, Märss T, Caldwell M, Wilson MVH. New and biostratigraphically useful thelodonts from the Silurian of the Mackenzie Mountains, Northwest Territories, Canada. Journal of Vertebrate Paleontology. 2001;21: 651–659.

11. Turner S. A new articulated thelodont (Agnatha) from the Early Devonian of Britain. Palaeontology. 1982;25: 879–89.

12. Wilson MVH, Caldwell MW. New Silurian and Devonian fork-tailed ‘thelodonts’ are jawless vertebrates with stomachs and deep bodies. Nature. 1993;361: 442–444.

13. Wilson MVH, Caldwell MW. The furcacaudiformes: A new order of jawless vertebrates with thelodont scales, based on articulated Silurian and Devonian fossils from northern Canada. Journal of Vertebrate Paleontology. 1998;18: 10–29.

14. Märss T, Turner S, Karatajûte−Talimaa V. Handbook of Palaeoichthyology, Volume 1B, Agnatha II. Verlag Dr. Friedrich Pfeil, Munchen; 2007.

15. Märss T., Karatajûte−Talimaa V. Ordovician and Lower Silurian thelodonts from Severnaya Zemlya Archipelago (Russia). Geodiversitas. 2002;24: 381–404.

16. Turner S, Nowlan GS. Early Silurian microvertebrates of eastern Canada. Bulletin du Muséum national d’Histoire naturelle, Paris. 1995;17: 513–29.

17. Sansom IJ, Elliott DK. A thelodont from the Ordovician of Canada. Journal of Vertebrate Paleontology. 2002;22: 867–870.

18. Aldridge RJ, Turner S, Jones GL, Harper DAT. Late Llandovery thelodonts and conodonts from the Kilbride Formation, Co. Galway, western Ireland. Geological Journal. 1996;31: 359­­­­–367.

19. Turner S. New Llandovery to early Pridoli microvertebrates including Lower Silurian zone fossil, *Loganellia avonia* nov. sp., from Britain. Courier-Forschungsinstitut Senckenberg. 2000; 91–128.

20. Blom H. Loganellia (Thelodonti, Agnatha) from the Lower Silurian of North Greenland. Acta Geologica Polonica. 1999;49: 97–104.

21. Karatajûte−Talimaa V. Silurian and Devonian thelodonts of the USSR and Spitsbergen. Translation Bureau; 1978.

22. Märss T. Thelodonts (Agnatha) from the basal beds of the Kuressaare Stage, Ludlow, Upper Silurian of Estonia. Proceedings of the Estonian Academy of Sciences, Geology. 2006;55: 43–66.

23. Märss T. *Loganellia* (Thelodonti, Agnatha) from the Jaagarahu Stage, Wenlock. Proceedings of the Estonian Academy of Sciences, Geology. 1996;45: 189–202.

24. Märss T, Miller GC. Thelodonts and distribution of associated conodonts from the Llandovery–lowermost Lochkovian of the Welsh Borderland. Palaeontology. 2004;47: 1211–1265.

25. Žigaitė Z. Endemic thelodonts (Vertebrata: Thelodonti) from the Lower Silurian of central Asia and southern Siberia. Earth and Environmental Science Transactions of the Royal Society of Edinburgh. 2013;104: 1–21.

26. Žigaitė Z. A new thelodont from Lower Silurian of Tuva and north-west Mongolia. Acta Universitatis Latviensis. 2004;679: 158–165.

27. Märss T. *Paralogania* from the Rootsikiila (Wenlock) and Paadla (Ludlow) stages of Estonia. Proceedings of the Estonian Academy of Sciences, Geology. 2003;52: 98–112.

28. Karatajûte−Talimaa V, Märss T. Upper Silurian thelodonts from Severnaya Zemlya Archipelago (Russia). Geodiversitas. 2002;24: 405–443.

29. Miller CG, Märss T. A conodont, thelodont and acanthodian fauna from the Lower Přidoli (Silurian) of the Much Wenlock Area, Shropshire. Palaeontology. 1999;42: 691–714.

30. Hairapetian V, Blom H, Miller CG. Silurian thelodonts from the Niur Formation, central Iran. Acta Palaeontologica Polonica. 2008;53: 85–95.

31. Turner S, Kuglitsch JJ, Clark DL. Llandoverian thelodont scales from the Burnt Bluff Group of Wisconsin and Michigan. Journal of Paleontology. 1999;73: 667–676.

32. Vergoossen JMJ. Late Silurian fish microfossils from Klinta and Rinnebäcks Bro (Scania, south Sweden), with remarks on the morphology of *Nostolepis striata* trunk scales. Scripta Geologica. 2002;123: 71–92.

33. Wang NZ. Restudy of thelodont microfossils from the lower part of the Cuifengshan Group of Qujing, eastern Yunnan, China. Vertebrata PalAsiatica. 1997;35: 1–17.

34. Wang NZ. Thelodont, acanthodian, and chondrichthyan fossils from the Lower Devonian of Southwest China. Proceedings of the Linnean Society of New South Wales. 1984;107: 419–441.

35. Märss T. A New Late Silurian or Early Devonian Thelodont from the Boothia Peninsula, Arctic Canada. Palaeontology. 1999;42: 1079–1099.

36. Botella H, Valenzuela‐Ríos JI, Carls P. A new Early Devonian thelodont from Celtiberia (Spain), with a revision of Spanish thelodonts. Palaeontology. 2006;49: 141–154.

37. Turner S, Young GC. Thelodont scales from the Middle-Late Devonian Aztec Siltstone, southern Victoria Land, Antarctica. Antarctic science. 1992;4: 89–105.

38. Blom H, Goujet D. Thelodont scales from the Lower Devonian Red Bay Group, Spitsbergen. Palaeontology. 2002;45: 795–820.

39. Karatajûte−Talimaa V. Lower Devonian (Lochkovian) thelodonts from the October Revolution Island (Severnaya Zemlya Archipelago, Russia). Geodiversitas. 2002;24: 791–804.

40. Burrow CJ, Turner S, Young GC. Middle Palaeozoic microvertebrate assemblages and biogeography of East Gondwana (Australasia, Antarctica). Palaeoworld. 2010;19: 37–54.

41. Turner S. Devonian thelodont scales (Agnatha, Thelodonti) from Queensland. Memoirs-Queensland Museum. 1995;38: 677–686.

42. Turner S, Jones PJ, Draper JJ. Early Devonian thelodonts (Agnatha) from the Toko Syncline, western Queensland, and a review of other Australian discoveries. BMR Journal of Australian Geology and Geophysics. 1981;6: 51–69.

43. Gagnier PY, Turner S, Friman L, Suarez-Riglos M, Janvier P. The Devonian vertebrate and mollusc fauna from Seripona (Dept. of Chuquisaca, Bolivia). Neues Jahrbuch für Geologie und Paläontologie Abhandlungen. 1988;176: 269–297.

44. Turner S, Burrow CJ, Gholamalian H, Yazdi M. Late Devonian (early Frasnian) microvertebrates and conodonts from the Chahriseh area near Esfahan, Iran. Memoirs of the Association of Australasian Palaeontologists. 2002;27: 149–159.

45. Wang ST, Gong ZZ, Turner S. Discovery of Middle Devonian Turiniidae (Thelodonti: Agnatha) from western Yunnan, China. Alcheringa. 1986;10: 315–325.

46. Hairapetian V, Roelofs BP, Trinajstic KM, Turner S. Famennian survivor turiniid thelodonts of North and East Gondwana. Geological Society, London, Special Publications. 2015;423: SP423. 3.

47. Turner S, Dring RS. Late Devonian thelodonts (Agnatha) from the Gneudna Formation, Carnarvon Basin, Western Australia. Alcheringa. 1981;5: 39–48.

48. Žigaitė‎ Z, Karatajûte−Talimaa V, Goujet D, Blom H. Thelodont scales from the Lower and Middle Devonian Andrée Land Group, Spitsbergen. 2013;135: 57–73.

49. Turner S. Siluro-Devonian thelodonts from the Welsh Borderland. Journal of the Geological Society. 1973;129: 557–582.

50. Märss T. Thelodont *Oeselia mosaica* gen. et sp. nov. from the Wenlock and Ludlow of the East Baltic. Proceedings of the Estonian Academy of Sciences, Geology. 2005;54: 181–190.
