# Supplementary material for: Squamation and ecology of thelodonts
Source: PLoS One. 2017 Feb 27;12(2):e0172781. doi: 10.1371/journal.pone.0172781 (PMC5328365; doi:10.1371/journal.pone.0172781)
Supplement: S1 Appendix — Compilation of all described species of thelodonts and the assigned ecological group with indication of body coverage percentages of each scale morphotype and functional type, details of the specimens preservation (CS, complete squamation; DR, desarticulated remains; PS, partial squamation; SP, scale patch; d, dorsal view; dv, dorsal and ventral view; v, ventral view; i, indeterminate view; l, lateral view) and type of assignment process (CVA, qualitative interpretation or both). Remains that allowed the ecological group assignment of each species are indicated in boldface. (DOCX) [file pone.0172781.s018.docx]

**S1 Appendix. Ecological diversity in thelodonts.**

Compilation of all described species of thelodonts and the assigned ecological group with indication of body coverage percentages of each scale morphotype and functional type, details of the specimens preservation (CS, complete squamation; DR, desarticulated remains; PS, partial squamation; SP, scale patch; d, dorsal view; dv, dorsal and ventral view; v, ventral view; i, indeterminate view; l, lateral view) and type of assignment process (CVA, qualitative interpretation or both). Remains that allowed the ecological group assignment of each species are indicated in boldface.

|  | **% of coverage of each funtional type and morphotype** | | | | | | | | **Preserv.** | **Assignment process** | **Ecological group** |
| --- | --- | --- | --- | --- | --- | --- | --- | --- | --- | --- | --- |
|  | **Bioluminescent** | | | **Defens.** | **Hydrod.** | **Abrasive** | | **General.** |  |  |  |
|  | **1** | **2** | **3** | **4** | **5** | **6** | **7** | **8** |  |  |  |
| **SANDIVIIFORMES** |  |  |  |  |  |  |  |  |  |  |  |
| **Sandiviidae** |  |  |  |  |  |  |  |  |  |  |  |
| *Sandivia melinkovi* | 0.0 | 0.0 | 0.0 | 0.0 | 0.0 | 100.0 | 0.0 | 0.0 | **DR** | CVA | Hard substrate |
| *Sandivia angusta* | 0.0 | 0.0 | 0.0 | 0.0 | 0.0 | 100.0 | 0.0 | 0.0 | **DR** | CVA | Hard substrate |
| **Stroinolepididae** |  |  |  |  |  |  |  |  |  |  |  |
| *Stroinolepis maenniki* | 0.0 | 0.0 | 0.0 | 0.0 | 0.0 | 100.0 | 0.0 | 0.0 | **DR** | CVA | Hard substrate |
| *Valyalepis crista* | 0.0 | 0.0 | 0.0 | 0.0 | 0.0 | 100.0 | 0.0 | 0.0 | **DR** | CVA | Hard substrate |
| **Angaralepididae** |  |  |  |  |  |  |  |  |  |  |  |
| *Angaralepis moskalenkoae* | 0.0 | 0.0 | 0.0 | 0.0 | 0.0 | 27.3 | 72.7 | 0.0 | **DR** | CVA | Hard substrate |
| ***incertae sedis*** |  |  |  |  |  |  |  |  |  |  |  |
| *Larolepis darbyi* | 0.0 | 0.0 | 0.0 | 0.0 | 0.0 | ? | 0.0 | 0.0 | DR | None | - |
|  |  |  |  |  |  |  |  |  |  |  |  |
| **LOGANELLIIFORMES** |  |  |  |  |  |  |  |  |  |  |  |
| **Loganelliidae** |  |  |  |  |  |  |  |  |  |  |  |
| *Loganellia scotica* | 0.0 | 0.0 | 0.0 | 0.0 | 0.0 | 36.8 | 0.0 | 63.2 | **CSdv** | Qualitative | Open water |
|  | 0.0 | 0.0 | 0.0 | 0.0 | 0.0 | 35.4 | 0.0 | 64.6 | CSd | - | - |
|  | 0.0 | 0.0 | 0.0 | 0.0 | 0.0 | 38.1 | 0.0 | 61.9 | **CSv** | CVA | Open water |
| *Loganellia aldridgei* | 0.0 | 0.0 | 0.0 | 0.0 | 0.0 | 4.6 | 0.0 | 95.4 | **DR** | CVA | Open water |
| *Loganellia almgreeni* | 0.0 | 0.0 | 0.0 | 0.0 | 0.0 | 27.3 | 72.7 | 0.0 | **DR** | CVA | Hard substrate |
| *Loganellia asiatica* | 0.0 | 0.0 | 0.0 | 0.0 | 0.0 | 27.3 | 72.7 | 0.0 | **DR** | CVA | Hard substrate |
| *Loganellia avonia* | 0.0 | 0.0 | 0.0 | 0.0 | 0.0 | 100.0 | 0.0 | 0.0 | **DR** | CVA | Hard substrate |
| *Loganellia cuneata* | 0.0 | 0.0 | 0.0 | 0.0 | 0.0 | ? | 0.0 | ? | **DR** | Qualitative | Soft substrate |
| *Loganellia einari* | 0.0 | 0.0 | 0.0 | 0.0 | 0.0 | 4.6 | 0.0 | 95.4 | **DR** | CVA | Open water |
| *Loganellia exilis* | 0.0 | 0.0 | 0.0 | 0.0 | 0.0 | ? | ? | ? | **DR** | Qualitative | Soft substrate |
| *Loganellia grossi* | 0.0 | 0.0 | 0.0 | 0.0 | 0.0 | 100.0 | 0.0 | 0.0 | **DR** | CVA | Hard substrate |
| *Loganellia incompta* | 0.0 | 0.0 | 0.0 | 0.0 | 0.0 | 100.0 | 0.0 | 0.0 | **DR** | CVA | Hard substrate |
| *Loganellia matura* | 0.0 | 0.0 | 0.0 | 0.0 | 0.0 | 95.4 | 4.6 | 0.0 | **DR** | CVA | Hard substrate |
| *Loganellia prolata* | 0.0 | 0.0 | 0.0 | 0.0 | 0.0 | 11.2 | 0.0 | 88.8 | **CSd** | CVA, qualitative | Open water or Soft substrate |
| *Loganellia sibirica* | 0.0 | 0.0 | 0.0 | 0.0 | 0.0 | 100.0 | 0.0 | 0.0 | **DR** | CVA | Hard substrate |
| *Loganellia sulcata* | 0.0 | 0.0 | 0.0 | 0.0 | 0.0 | 14.4 | 0.0 | 85.6 | CSd | - | - |
|  | 0.0 | 0.0 | 0.0 | 0.0 | 0.0 | 0.0 | 0.0 | 100.0 | **CSv** | CVA | Open water |
| *Loganellia tuvaensis* | 0.0 | 0.0 | 0.0 | 0.0 | 0.0 | 100.0 | 0.0 | 0.0 | **DR** | CVA | Hard substrate |
| *Loganellia unispinata* | 0.0 | 0.0 | 0.0 | 0.0 | 0.0 | ? | 0.0 | ? | **DR** | Qualitative | Soft substrate |
| *Illoganellia colossea* | 0.0 | 0.0 | 0.0 | 0.0 | 0.0 | 0.0 | 100.0 | 0.0 | **SP** | None | - |
| *Talimaalepis rimae* | 0.0 | 0.0 | 0.0 | 0.0 | 0.0 | 4.6 | 0.0 | 95.4 | **DR** | CVA | Open water |
| *Talimaalepis kadvoiensis* | 0.0 | 0.0 | 0.0 | 0.0 | 0.0 | 4.6 | 0.0 | 95.4 | **DR** | CVA | Open water |
| **Nunavutiidae** |  |  |  |  |  |  |  |  |  |  |  |
| *Nunavutia fasciata* | 0.0 | 0.0 | 0.0 | 0.0 | 0.0 | 0.0 | 0.0 | ? | **DR** | Qualitative | Open water |
|  |  |  |  |  |  |  |  |  |  |  |  |
| **SHIELIIFORMES** |  |  |  |  |  |  |  |  |  |  |  |
| **Shieliidae** |  |  |  |  |  |  |  |  |  |  |  |
| *Shielia taiti* | 0.0 | 0.0 | 0.0 | 90.4 | 0.0 | 9.6 | 0.0 | 0.0 | **CSdv** | CVA | Schooling |
|  | 0.0 | 0.0 | 0.0 | 92.6 | 0.0 | 7.4 | 0.0 | 0.0 | **CSd** | CVA | Schooling |
|  | 0.0 | 0.0 | 0.0 | 88.2 | 0.0 | 11.8 | 0.0 | 0.0 | **CSv** | CVA | Schooling |
| *Shielia gibba* | 0.0 | 0.0 | 0.0 | 100.0 | 0.0 | 0.0 | 0.0 | 0.0 | **CSi** | CVA | Schooling |
|  | 0.0 | 0.0 | 0.0 | ? | 0.0 | 0.0 | 0.0 | 0.0 | **DR** | Qualitative | Schooling |
| *Shielia multispinata* | 0.0 | 0.0 | 0.0 | 72.7 | 0.0 | 27.3 | 0.0 | 0.0 | **DR** | CVA | Schooling |
| *Shielia parca* | 0.0 | 0.0 | 0.0 | 92.2 | 0.0 | 7.8 | 0.0 | 0.0 | **CSd** | CVA | Schooling |
|  | 0.0 | 0.0 | 0.0 | 65.4 | 0.0 | 34.6 | 0.0 | 0.0 | **DR** | CVA | Schooling |
| *Paralogania kummerowi* | 0.0 | 0.0 | 0.0 | 0.0 | 0.0 | 100.0 | 0.0 | 0.0 | **DR** | CVA | Hard substrate |
| *Paralogania borealis* | 0.0 | 0.0 | 0.0 | 0.0 | 0.0 | 100.0 | 0.0 | 0.0 | **DR** | CVA | Hard substrate |
| *Paralogania consimilis* | 0.0 | 0.0 | 0.0 | 72.7 | 0.0 | 27.3 | 0.0 | 0.0 | **DR** | CVA | Schooling |
| *Paralogania* ex gr*. martinssoni* | 0.0 | 0.0 | 0.0 | 72.7 | 0.0 | 27.3 | 0.0 | 0.0 | **DR** | CVA | Schooling |
| *Paralogania foliala* | 0.0 | 0.0 | 0.0 | 0.0 | 0.0 | 100.0 | 0.0 | 0.0 | **DR** | CVA | Hard substrate |
| *Paralogania kaarmisensis* | 0.0 | 0.0 | 0.0 | 72.7 | 0.0 | 27.3 | 0.0 | 0.0 | **DR** | CVA | Schooling |
| *Paralogania kachanovi* | 0.0 | 0.0 | 0.0 | 0.0 | 0.0 | 100.0 | 0.0 | 0.0 | **DR** | CVA | Hard substrate |
| *Paralogania klubovi* | 0.0 | 0.0 | 0.0 | ? | 0.0 | ? | 0.0 | 0.0 | **DR** | Qualitative | Schooling |
| *Paralogania ludlowiensis* | 0.0 | 0.0 | 0.0 | 100.0 | 0.0 | 0.0 | 0.0 | 0.0 | **DR** | CVA | Schooling |
| *Paralogania martinssoni* | 0.0 | 0.0 | 0.0 | 0.0 | 0.0 | 0.0 | 4.6 | 95.4 | **DR** | CVA | Open water |
| *Paralogania menneri* | 0.0 | 0.0 | 0.0 | ? | 0.0 | ? | 0.0 | 0.0 | **DR** | Qualitative | Schooling |
| *Paralogania perensae* | 0.0 | 0.0 | 0.0 | 72.7 | 0.0 | 27.3 | 0.0 | 0.0 | **DR** | CVA | Schooling |
| *Paralogania readbayensis* | 0.0 | 0.0 | 0.0 | 72.7 | 0.0 | 22.7 | 4.6 | 0.0 | **DR** | CVA | Schooling |
| *Paralogania tarranti* | 0.0 | 0.0 | 0.0 | 0.0 | 0.0 | 100.0 | 0.0 | 0.0 | **DR** | CVA | Hard substrate |
| *Paralogania wilsoni* | 0.0 | 0.0 | 0.0 | 0.0 | 0.0 | 100.0 | 0.0 | 0.0 | **DR** | CVA | Hard substrate |
| *Valiukia flabellata* | 0.0 | 0.0 | 0.0 | 0.0 | 0.0 | 27.3 | 72.7 | 0.0 | **DR** | CVA | Hard substrate |
| *Nethertonodus prodigialis* | 0.0 | 0.0 | 0.0 | 72.7 | 0.0 | 27.3 | 0.0 | 0.0 | **DR** | CVA | Schooling |
| *Nethertonodus laadjalaensis* | 0.0 | 0.0 | 0.0 | 0.0 | 0.0 | 0.0 | 0.0 | ? | **DR** | Qualitative | Open water |
| *Praetrilogania grabion* | 0.0 | 0.0 | 0.0 | 0.0 | 72.7 | 27.3 | 0.0 | 0.0 | **DR** | CVA | Strong-swimming |
|  |  |  |  |  |  |  |  |  |  |  |  |
| **PHLEBOLEPIDIFORMES** |  |  |  |  |  |  |  |  |  |  |  |
| **Phlebolepididae** |  |  |  |  |  |  |  |  |  |  |  |
| *Phlebolepis elegans* | 0.0 | 0.0 | 0.0 | 0.0 | 0.0 | 0.0 | 100.0 | 0.0 | **CSl** | CVA | Hard substrate |
| *Phlebolepis ornata* | 0.0 | 0.0 | 0.0 | 0.0 | 0.0 | 27.3 | 72.7 | 0.0 | **DR** | CVA | Hard substrate |
| *Erepsilepis margaritifera* | 0.0 | 0.0 | 0.0 | 0.0 | 0.0 | 0.0 | 100.0 | 0.0 | **CSi** | CVA | Hard substrate or Soft substrate |
| *Helenolepis obruchevi* | 0.0 | 0.0 | 0.0 | 0.0 | 0.0 | ? | 0.0 | 0.0 | **DR** | Qualitative | Hard substrate |
| *Helenolepis multicostata* | 0.0 | 0.0 | 0.0 | 0.0 | 0.0 | 0.0 | ? | 0.0 | **DR** | Qualitative | Hard substrate |
| *Helenolepis navicularis* | 0.0 | 0.0 | 0.0 | 0.0 | 0.0 | 100.0 | 0.0 | 0.0 | **DR** | CVA | Hard substrate |
| **Katoporididae** |  |  |  |  |  |  |  |  |  |  |  |
| *Trimerolepis lithuanica* | 0.0 | 0.0 | 0.0 | ? | 0.0 | ? | 0.0 | 0.0 | **DR** | Qualitative | Schooling |
| *Trimerolepis gemella* | 0.0 | 0.0 | 0.0 | 0.0 | 72.7 | 27.3 | 0.0 | 0.0 | **DR** | CVA | Strong-swimming |
| *Trimerolepis serrata* | 0.0 | 0.0 | 0.0 | 72.7 | 0.0 | 27.3 | 0.0 | 0.0 | **DR** | CVA | Schooling |
| *Trimerolepis timanica* | 0.0 | 0.0 | 0.0 | 0.0 | 0.0 | 100.0 | 0.0 | 0.0 | **DR** | CVA | Hard substrate |
| *Trimerolepis tricava* | 0.0 | 0.0 | 0.0 | 72.7 | 0.0 | 27.3 | 0.0 | 0.0 | **DR** | CVA | Schooling |
| *Goniporus alatus* | 0.0 | 0.0 | 0.0 | 0.0 | 0.0 | 100.0 | 0.0 | 0.0 | **DR** | CVA | Hard substrate |
| *Niurolepis susanae* | 0.0 | 0.0 | 0.0 | 72.7 | 0.0 | 22.7 | 4.6 | 0.0 | **DR** | CVA | Schooling |
| *Zueguelepis potanus* | 0.0 | 0.0 | 0.0 | 72.7 | 0.0 | 0.0 | 27.3 | 0.0 | **DR** | CVA | Schooling |
| *Overia adraini* | 0.0 | 0.0 | 0.0 | 0.0 | 0.0 | ? | ? | ? | **DR** | Qualitative | Soft substrate |
|  |  |  |  |  |  |  |  |  |  |  |  |
| **THELODONTIFORMES** |  |  |  |  |  |  |  |  |  |  |  |
| **Thelodontididae** |  |  |  |  |  |  |  |  |  |  |  |
| *Thelodus parvidens* | 0.0 | 0.0 | 0.0 | 0.0 | 0.0 | 100.0 | 0.0 | 0.0 | **DR** | CVA | Hard substrate |
| *Thelodus admirabilis* | 0.0 | 0.0 | 0.0 | 0.0 | 0.0 | ? | 0.0 | 0.0 | DR | None | - |
| *Thelodus calvus* | 0.0 | 0.0 | 0.0 | 0.0 | 0.0 | ? | 0.0 | 0.0 | **DR** | Qualitative | Hard substrate |
| *Thelodus carinatus* | 0.0 | 0.0 | 0.0 | 0.0 | 0.0 | ? | ? | ? | **DR** | Qualitative | Soft substrate |
| *Thelodus inauditus* | 0.0 | 0.0 | 0.0 | 0.0 | 0.0 | 100.0 | 0.0 | 0.0 | SP | None | - |
| *Thelodus laevis* | 0.0 | 0.0 | 0.0 | 0.0 | 0.0 | ? | 0.0 | ? | PSi | None | - |
| *Thelodus laevis* | 0.0 | 0.0 | 0.0 | 0.0 | 0.0 | ? | 0.0 | ? | DR | None | - |
| *Thelodus macintoshi* | 0.0 | 0.0 | 0.0 | 0.0 | 0.0 | 100.0 | 0.0 | 0.0 | PSi | Qualitative | Hard substrate or Soft substrate |
| *Thelodus marginatus* | 0.0 | 0.0 | 0.0 | 0.0 | 0.0 | ? | 0.0 | ? | **DR** | Qualitative | Soft substrate |
| *Thelodus matukhini* | 0.0 | 0.0 | 0.0 | 0.0 | 0.0 | ? | ? | ? | **DR** | Qualitative | Soft substrate |
| *Thelodus sculptilis* | 0.0 | 0.0 | 0.0 | 0.0 | 0.0 | 100.0 | 0.0 | 0.0 | **DR** | CVA | Hard substrate |
| *Thelodus traquairi* | 0.0 | 0.0 | 0.0 | 0.0 | 0.0 | 0.0 | ? | 0.0 | **DR** | Qualitative | Hard substrate |
| *Thelodus trilobatus* | 0.0 | 0.0 | 0.0 | 0.0 | 0.0 | 4.6 | 0.0 | 95.4 | **DR** | CVA | Open water |
| *Thelodus visvaldi* | 0.0 | 0.0 | 0.0 | 0.0 | 72.7 | 27.3 | 0.0 | 0.0 | **DR** | CVA | Strong-swimming |
| *Parathelodus scitulus* | 0.0 | 0.0 | 0.0 | 0.0 | 0.0 | ? | 0.0 | 0.0 | **DR** | Qualitative | Hard substrate |
| *Parathelodus asiaticus* | 0.0 | 0.0 | 0.0 | 0.0 | 0.0 | 100.0 | 0.0 | 0.0 | **DR** | CVA | Hard substrate |
| *Parathelodus catalatus* | 0.0 | 0.0 | 0.0 | 0.0 | 0.0 | ? | 0.0 | 0.0 | **DR** | Qualitative | Hard substrate |
| *Parathelodus cornuformis* | 0.0 | 0.0 | 0.0 | 0.0 | 0.0 | ? | 0.0 | 0.0 | **DR** | Qualitative | Hard substrate |
| *Parathelodus trilobatus* | 0.0 | 0.0 | 0.0 | 0.0 | 0.0 | ? | 0.0 | 0.0 | **DR** | Qualitative | Hard substrate |
| **Archipelepididae** |  |  |  |  |  |  |  |  |  |  |  |
| *Archipelepis turbinata* | 0.0 | 0.0 | 0.0 | 0.0 | 0.0 | 0.0 | 100.0 | 0.0 | PSi | - | - |
|  | 0.0 | 0.0 | 0.0 | 0.0 | 0.0 | 0.0 | ? | 0.0 | **DR** | Qualitative | Hard substrate |
| *Archipelepis bifurcata* | 0.0 | 0.0 | 0.0 | 0.0 | 0.0 | 0.0 | 100.0 | 0.0 | **PSi** | Qualitative | Hard substrate or Soft substrate |
|  | 0.0 | 0.0 | 0.0 | 0.0 | 0.0 | 0.0 | ? | 0.0 | DR | - | Hard substrate or Soft substrate |
| **Boothialepididae** |  |  |  |  |  |  |  |  |  |  |  |
| *Boothialepis throsteinssoni* | 0.0 | 0.0 | 0.0 | 0.0 | 0.0 | 4.6 | 95.4 | 0.0 | **DR** | CVA | Hard substrate |
| **Eestilepididae** |  |  |  |  |  |  |  |  |  |  |  |
| *Eestilepis prominens* | 0.0 | 0.0 | 0.0 | 0.0 | 0.0 | 100.0 | 0.0 | 0.0 | **PSdv** | Qualitative | Hard substrate |
| **Lanarkiidae** |  |  |  |  |  |  |  |  |  |  |  |
| *Lanarkia horrida* | 0.0 | 0.0 | 0.0 | 0.0 | 0.0 | 10.0 | 0.0 | 90.0 | **CSdv** | CVA | Open water |
|  | 0.0 | 0.0 | 0.0 | 0.0 | 0.0 | 9.6 | 0.0 | 90.5 | **CSd** | - | - |
|  | 0.0 | 0.0 | 0.0 | 0.0 | 0.0 | 10.4 | 0.0 | 89.6 | **CSv** | CVA | Open water |
| *Lanarkia lanceolata* | 0.0 | 0.0 | 0.0 | 0.0 | 0.0 | 6.7 | 0.0 | 93.3 | **CSi** | CVA, qualitative | Open water or Soft substrate |
| *Lanarkia spinulosa* | 0.0 | 0.0 | 0.0 | 100.0 | 0.0 | 0.0 | 0.0 | 0.0 | **PSi** | Qualitative | Schooling |
| *Phillipsilepis crassa* | 0.0 | 0.0 | 0.0 | 0.0 | 0.0 | 0.0 | 100.0 | 0.0 | **CSv** | CVA, qualitative | Hard substrate or Soft substrate |
| *Phillipsilepis cornuta* | 0.0 | 0.0 | 0.0 | 0.0 | 0.0 | 0.0 | 100.0 | 0.0 | **PSi** | Qualitative | Hard substrate or Soft substrate |
| *Phillipsilepis pusilla* | 0.0 | 0.0 | 0.0 | 0.0 | 0.0 | 0.0 | 100.0 | 0.0 | **CSi** | CVA, qualitative | Hard substrate or Soft substrate |
| **Turiniidae** |  |  |  |  |  |  |  |  |  |  |  |
| *Turinia pagei* | 0.0 | 0.0 | 0.0 | 0.0 | 0.0 | 100.0 | 0.0 | 0.0 | CSv | - | - |
| *Turinia pagei* | 0.0 | 0.0 | 0.0 | 0.0 | 0.0 | 100.0 | 0.0 | 0.0 | **DR** | CVA | Hard substrate |
| *Turinia antarctica* | 0.0 | 0.0 | 0.0 | 0.0 | 0.0 | 100.0 | 0.0 | 0.0 | **DR** | CVA | Hard substrate |
| *Turinia australiensis* | 0.0 | 0.0 | 0.0 | 0.0 | 0.0 | 100.0 | 0.0 | 0.0 | **DR** | CVA | Hard substrate |
| *Turinia barentsia* | 0.0 | 0.0 | 0.0 | 0.0 | 0.0 | ? | 0.0 | 0.0 | **DR** | Qualitative | Hard substrate |
| *Turinia composita* | 0.0 | 0.0 | 0.0 | 0.0 | 0.0 | ? | 0.0 | ? | **DR** | Qualitative | Soft substrate |
| *Turinia fuscina* | 0.0 | 0.0 | 0.0 | ? | 0.0 | 0.0 | 0.0 | 0.0 | **DR** | Qualitative | Schooling |
| *Turinia gavinyoungi* | 0.0 | 0.0 | 0.0 | 72.7 | 0.0 | 0.0 | 27.3 | 0.0 | **DR** | CVA | Schooling |
| *Turinia gondwana* | 0.0 | 0.0 | 0.0 | 0.0 | 0.0 | ? | ? | ? | **DR** | Qualitative | Soft substrate |
| *Turinia hutkensis* | 0.0 | 0.0 | 0.0 | 0.0 | 0.0 | 100.0 | 0.0 | 0.0 | **DR** | CVA | Hard substrate |
| *Turinia nachoi* | 0.0 | 0.0 | 0.0 | 72.7 | 0.0 | 0.0 | 27.3 | 0.0 | **DR** | CVA | Schooling |
| *Turinia pagoda* | 0.0 | 0.0 | 0.0 | 0.0 | 0.0 | ? | 0.0 | 0.0 | **DR** | Qualitative | Hard substrate |
| *Turinia polita* | 0.0 | 0.0 | 0.0 | 0.0 | 0.0 | 100.0 | 0.0 | 0.0 | **DR** | CVA | Hard substrate |
| *Arianalepis megacostata* | 0.0 | 0.0 | 0.0 | 0.0 | 0.0 | ? | ? | 0.0 | **DR** | Qualitative | Hard substrate |
| *Australolepis seddoni* | 0.0 | 0.0 | 0.0 | 95.4 | 0.0 | 4.6 | 0.0 | 0.0 | **DR** | CVA | Schooling |
| *Jesslepis johnsoni* | 0.0 | 0.0 | 0.0 | 0.0 | 0.0 | 100.0 | 0.0 | 0.0 | **DR** | CVA | Hard substrate |
| *Boreania minima* | 0.0 | 0.0 | 0.0 | 0.0 | 0.0 | 100.0 | 0.0 | 0.0 | **DR** | CVA | Hard substrate |
| *Woodfjordia collisa* | 0.0 | 0.0 | 0.0 | 0.0 | 0.0 | ? | ? | 0.0 | **DR** | Qualitative | Hard substrate |
| **Nikoliviidae** |  |  |  |  |  |  |  |  |  |  |  |
| *Nikolivia milesi* | 0.0 | 0.0 | 0.0 | 43.8 | 0.0 | 56.3 | 0.0 | 0.0 | **SP** | Qualitative | Schooling |
| **Barlowodide** |  |  |  |  |  |  |  |  |  |  |  |
| *Barlowodus excelsus* | 0.0 | 0.0 | 0.0 | 72.7 | 0.0 | 27.3 | 0.0 | 0.0 | **DR** | CVA | Schooling |
| *Barlowodus floralis* | 0.0 | 0.0 | 0.0 | 72.7 | 0.0 | 27.3 | 0.0 | 0.0 | **DR** | CVA | Schooling |
| *Barlowodus tridens* | 0.0 | 0.0 | 0.0 | 0.0 | 0.0 | 0.0 | 0.0 | ? | **DR** | Qualitative | Open water |
| **Nikoliviidae** |  |  |  |  |  |  |  |  |  |  |  |
| *Nikolivia oervigi* | 0.0 | 0.0 | 0.0 | ? | 0.0 | 0.0 | 0.0 | 0.0 | **DR** | Qualitative | Schooling |
| *Nikolivia aligera* | 0.0 | 0.0 | 0.0 | ? | 0.0 | ? | 0.0 | 0.0 | **DR** | Qualitative | Schooling |
| *Nikolivia auriculata* | 0.0 | 0.0 | 0.0 | 72.7 | 0.0 | 0.0 | 27.3 | 0.0 | **DR** | CVA | Schooling |
| *Nikolivia balabayi* | 0.0 | 0.0 | 0.0 | ? | 0.0 | ? | 0.0 | 0.0 | **DR** | Qualitative | Schooling |
| *Nikolivia depressa* | 0.0 | 0.0 | 0.0 | 0.0 | 0.0 | 100.0 | 0.0 | 0.0 | **DR** | CVA | Hard substrate |
| *Nikolivia gutta* | 0.0 | 0.0 | 0.0 | 0.0 | 0.0 | 100.0 | 0.0 | 0.0 | **DR** | CVA | Hard substrate |
| *Chattertonodus cometoides* | 0.0 | 0.0 | 0.0 | ? | 0.0 | ? | 0.0 | 0.0 | **DR** | Qualitative | Schooling |
| **Talivaliidae** |  |  |  |  |  |  |  |  |  |  |  |
| *Talivalia elongata* | 0.0 | 0.0 | 0.0 | 100.0 | 0.0 | 0.0 | 0.0 | 0.0 | **DR** | CVA | Schooling |
| *Talivalia svalbardia* | 0.0 | 0.0 | 0.0 | 0.0 | 0.0 | 0.0 | 4.6 | 95.4 | **DR** | CVA | Open water |
| *Glacialepis corpulenta* | 0.0 | 0.0 | 0.0 | ? | 0.0 | ? | 0.0 | 0.0 | **DR** | Qualitative | Schooling |
| *Amaltheolepis winsnesi* | 0.0 | 0.0 | 0.0 | 95.4 | 0.0 | 4.6 | 0.0 | 0.0 | **DR** | CVA | Schooling |
| *Amaltheolepis austfjordia* | 0.0 | 0.0 | 0.0 | 95.4 | 0.0 | 4.6 | 0.0 | 0.0 | **DR** | CVA | Schooling |
| *Amaltheolepis baltica* | 0.0 | 0.0 | 0.0 | ? | 0.0 | 0.0 | 0.0 | 0.0 | **DR** | Qualitative | Schooling |
| *Amaltheolepis bystrovi* | 0.0 | 0.0 | 0.0 | ? | 0.0 | 0.0 | ? | 0.0 | **DR** | Qualitative | Schooling |
| *Amaltheolepis montiwatsonia* | 0.0 | 0.0 | 0.0 | 0.0 | 0.0 | 0.0 | 27.3 | 72.7 | **DR** | CVA | Open water |
| **Apalolepididae** |  |  |  |  |  |  |  |  |  |  |  |
| *Apalolepis obruchevi* | 0.0 | 0.0 | 0.0 | 0.0 | 72.7 | 27.3 | 0.0 | 0.0 | **DR** | CVA | Strong-swimming |
| *Apalolepis angelica* | 0.0 | 0.0 | 0.0 | 0.0 | ? | 0.0 | 0.0 | 0.0 | **DR** | Qualitative | Strong-swimming |
| *Apalolepis brotzeni* | 0.0 | 0.0 | 0.0 | 0.0 | 72.7 | 27.3 | 0.0 | 0.0 | **DR** | CVA | Strong-swimming |
| *Apalolepis toombsi* | 0.0 | 0.0 | 0.0 | 0.0 | 0.0 | ? | 0.0 | 0.0 | DR | None | - |
| *Gampsolepis insueta* | 0.0 | 0.0 | 0.0 | ? | 0.0 | ? | 0.0 | 0.0 | **DR** | Qualitative | Schooling |
| ***incertae sedis*** |  |  |  |  |  |  |  |  |  |  |  |
| *Skamolepis fragilis* | 0.0 | 0.0 | 0.0 | 0.0 | ? | 0.0 | 0.0 | 0.0 | **DR** | Qualitative | Strong-swimming |
|  |  |  |  |  |  |  |  |  |  |  |  |
| **FURCACAUDIFORMES** |  |  |  |  |  |  |  |  |  |  |  |
| **Furcacaudidae** |  |  |  |  |  |  |  |  |  |  |  |
| *Canonia grossi* | 0.0 | 0.0 | 0.0 | 0.0 | ? | 0.0 | 0.0 | 0.0 | **DR** | Qualitative | Strong-swimming |
| *Canonia costulata* | 0.0 | 0.0 | 0.0 | 0.0 | 95.4 | 0.0 | 4.6 | 0.0 | **DR** | CVA | Strong-swimming |
| *Canonia kaerberi* | 0.0 | 0.0 | 0.0 | 0.0 | ? | 0.0 | 0.0 | 0.0 | **DR** | Qualitative | Strong-swimming |
| *Furcacauda heintzae* | 0.0 | 0.0 | 0.0 | 0.0 | 0.0 | 100.0 | 0.0 | 0.0 | **CSl** | CVA | Hard substrate |
| *Furcacauda fredholmae* | 0.0 | 0.0 | 0.0 | 4.6 | 0.0 | 95.4 | 0.0 | 0.0 | **CSl** | CVA | Hard substrate |
| *Cometicercus talimaae* | 0.0 | 0.0 | 0.0 | 100.0 | 0.0 | 0.0 | 0.0 | 0.0 | **CSl** | CVA | Schooling |
| *Drepanolepis maerssae* | 0.0 | 0.0 | 0.0 | 100.0 | 0.0 | 0.0 | 0.0 | 0.0 | **CSl** | CVA | Schooling |
| *Sphenonectris turnerae* | 0.0 | 0.0 | 0.0 | 90.7 | 0.0 | 0.0 | 9.3 | 0.0 | **CSl** | CVA | Schooling |
| **Pezopallichthydae** |  |  |  |  |  |  |  |  |  |  |  |
| *Pezopallichthys ritchiei* | 0.0 | 0.0 | 0.0 | 98.1 | 0.0 | 1.9 | 0.0 | 0.0 | **CSl** | CVA | Schooling |
|  |  |  |  |  |  |  |  |  |  |  |  |
| ***INCERTAE SEDIS*** |  |  |  |  |  |  |  |  |  |  |  |
| **Oeseliidae** |  |  |  |  |  |  |  |  |  |  |  |
| *Oeselia mosaica* | 0.0 | 0.0 | 0.0 | 0.0 | 0.0 | 100.0 | 0.0 | 0.0 | **DR** | CVA | Hard substrate |
| **Longodidae** |  |  |  |  |  |  |  |  |  |  |  |
| *Longodus acicularis* | 0.0 | 95.4 | 4.6 | 0.0 | 0.0 | 0.0 | 0.0 | 0.0 | **DR** | CVA, qualitative | - |
| ***incertae sedis*** |  |  |  |  |  |  |  |  |  |  |  |
| *Thulolepis striaspina* | 0.0 | 0.0 | 0.0 | 0.0 | 0.0 | 100.0 | 0.0 | 0.0 | **DR** | CVA | Hard substrate |
| *Sophialepis ancorata* | 0.0 | 0.0 | 0.0 | 0.0 | 0.0 | 100.0 | 0.0 | 0.0 | **DR** | CVA | Hard substrate |
